# Association of Adverse Outcomes With Emotion Processing and Its Neural Substrate in Individuals at Clinical High Risk for Psychosis

**DOI:** 10.1001/jamapsychiatry.2019.3501

**Published:** 2019-11-13

**Authors:** Gemma Modinos, Matthew J. Kempton, Stefania Tognin, Maria Calem, Lilla Porffy, Mathilde Antoniades, Ava Mason, Matilda Azis, Paul Allen, Barnaby Nelson, Patrick McGorry, Christos Pantelis, Anita Riecher-Rössler, Stefan Borgwardt, Rodrigo Bressan, Neus Barrantes-Vidal, Marie-Odile Krebs, Merete Nordentoft, Birte Glenthøj, Stephan Ruhrmann, Gabriele Sachs, Bart Rutten, Jim van Os, Lieuwe de Haan, Eva Velthorst, Mark van der Gaag, Lucia R. Valmaggia, Philip McGuire

**Affiliations:** 1 Department of Psychosis Studies, Institute of Psychiatry, Psychology & Neuroscience, King's College London, London, United Kingdom; 2 Department of Neuroimaging, Institute of Psychiatry, Psychology & Neuroscience, King's College London, London, United Kingdom; 3 National Institute for Health Research, Biomedical Research Centre, London, United Kingdom; 4 Department of Psychology, University of Roehampton, London, United Kingdom; 5 Orygen, The National Centre of Excellence in Youth Mental Health, University of Melbourne, Melbourne, Victoria, Australia; 6 Centre for Youth Mental Health, University of Melbourne, Melbourne, Victoria, Australia; 7 Center for Neuropsychiatric Schizophrenia Research, University of Copenhagen, Mental Health Centre Glostrup, Copenhagen, Denmark; 8 Center for Clinical Intervention and Neuropsychiatric Schizophrenia Research, University of Copenhagen, Mental Health Centre Glostrup, Copenhagen, Denmark; 9 Department of Clinical Medicine, Faculty of Health and Medical Sciences, University of Copenhagen, Copenhagen, Denmark; 10 University of Basel Psychiatric Hospital, Basel, Switzerland; 11 LiNC—Lab Interdisciplinar Neurociências Clínicas, Depto Psiquiatria, Escola Paulista de Medicina, Universidade Federal de São Paulo—UNIFESP, São Paulo, Brazil; 12 Departament de Psicologia Clínica i de la Salut (Universitat Autònoma de Barcelona), Fundació Sanitària Sant Pere Claver (Spain), Spanish Mental Health Research Network (CIBERSAM), Barcelona, Spain; 13 University of Paris, GHU-Paris, Sainte-Anne, C’JAAD, Hospitalo-Universitaire Department SHU, Inserm U1266, Institut de Psychiatrie (CNRS 3557), Paris, France; 14 Mental Health Center Copenhagen, Center for Clinical Intervention and Neuropsychiatric Schizophrenia Research, Mental Health Center Glostrup, Mental Health Services in the Capital Region of Copenhagen, University of Copenhagen, Copenhagen, Denmark; 15 Center for Clinical Intervention and Neuropsychiatric Schizophrenia Research, Mental Health Center Glostrup, Mental Health Services in the Capital Region of Copenhagen, University of Copenhagen, Copenhagen, Denmark; 16 Department of Psychiatry and Psychotherapy, University of Cologne, Cologne, Germany; 17 Department of Psychiatry and Psychotherapy, Medical University of Vienna, Vienna, Austria; 18 School for Mental Health and Neuroscience, Department of Psychiatry and Neuropsychology, Faculty of Health, Medicine and Life Sciences, Maastricht University, Maastricht, the Netherlands; 19 University Medical Centre Utrecht Brain Center, Department of Psychiatry, Utrecht University Medical Centre, Utrecht, the Netherlands; 20 Early Psychosis Department, Amsterdam UMC, Amsterdam, the Netherlands; 21 Department of Psychiatry, Icahn School of Medicine at Mount Sinai, New York, New York; 22 Amsterdam Public Mental Health Research Institute, Department of Clinical Psychology, Faculty of Behavioural and Movement Sciences, Vrije Universiteit Amsterdam, Amsterdam, the Netherlands; 23 Parnassia Psychiatric Institute, Department of Psychosis Research, The Hague, the Netherlands; 24 Institute of Psychiatry, Psychology & Neuroscience, Department of Psychology, King's College London, London, United Kingdom; 25 South London and Maudsley National Health Service Foundation Trust, London, United Kingdom

## Abstract

**Importance:**

The development of adverse clinical outcomes in patients with psychosis has been associated with behavioral and neuroanatomical deficits related to emotion processing. However, the association between alterations in brain regions subserving emotion processing and clinical outcomes remains unclear.

**Objective:**

To examine the association between alterations in emotion processing and regional gray matter volumes in individuals at clinical high risk (CHR) for psychosis, and the association with subsequent clinical outcomes.

**Design, Setting, and Participants:**

This naturalistic case-control study with clinical follow-up at 12 months was conducted from July 1, 2010, to August 31, 2016, and collected data from 9 psychosis early detection centers (Amsterdam, Basel, Cologne, Copenhagen, London, Melbourne, Paris, The Hague, and Vienna). Participants (213 individuals at CHR and 52 healthy controls) were enrolled in the European Network of National Schizophrenia Networks Studying Gene-Environment Interactions (EU-GEI) project. Data were analyzed from October 1, 2018, to April 24, 2019.

**Main Measures and Outcomes:**

Emotion recognition was assessed with the Degraded Facial Affect Recognition Task. Three-Tesla magnetic resonance imaging scans were acquired from all participants, and gray matter volume was measured in regions of interest (medial prefrontal cortex, amygdala, hippocampus, and insula). Clinical outcomes at 12 months were evaluated for transition to psychosis using the Comprehensive Assessment of At-Risk Mental States criteria, and the level of overall functioning was measured through the Global Assessment of Functioning [GAF] scale.

**Results:**

A total of 213 individuals at CHR (105 women [49.3%]; mean [SD] age, 22.9 [4.7] years) and 52 healthy controls (25 women [48.1%]; mean [SD] age, 23.3 [4.0] years) were included in the study at baseline. At the follow-up within 2 years of baseline, 44 individuals at CHR (20.7%) had developed psychosis and 169 (79.3%) had not. Of the individuals at CHR reinterviewed with the GAF, 39 (30.0%) showed good overall functioning (GAF score, ≥65), whereas 91 (70.0%) had poor overall functioning (GAF score, <65). Within the CHR sample, better anger recognition at baseline was associated with worse functional outcome (odds ratio [OR], 0.88; 95% CI, 0.78-0.99; *P* = .03). In individuals at CHR with a good functional outcome, positive associations were found between anger recognition and hippocampal volume (*z*e = 3.91; familywise error [FWE] *P* = .02) and between fear recognition and medial prefrontal cortex volume (*z* = 3.60; FWE *P* = .02), compared with participants with a poor outcome. The onset of psychosis was not associated with baseline emotion recognition performance (neutral OR, 0.93; 95% CI, 0.79-1.09; *P* = .37; happy OR, 1.03; 95% CI, 0.84-1.25; *P* = .81; fear OR, 0.98; 95% CI, 0.85-1.13; *P* = .77; anger OR, 1.00; 95% CI, 0.89-1.12; *P* = .96). No difference was observed in the association between performance and regional gray matter volumes in individuals at CHR who developed or did not develop psychosis (FWE *P* < .05).

**Conclusions and Relevance:**

In this study, poor functional outcome in individuals at CHR was found to be associated with baseline abnormalities in recognizing negative emotion. This finding has potential implications for the stratification of individuals at CHR and suggests that interventions that target socioemotional processing may improve functional outcomes.

## Key Points

**Question:**

Is altered emotion recognition associated with adverse clinical and functional outcomes in people at clinical high risk for psychosis?

**Findings:**

In this case-control study of 213 individuals at clinical high risk for psychosis and 52 healthy participants, abnormalities in the recognition of negative emotion at baseline were associated with neuroanatomical alterations in the medial prefrontal cortex and hippocampus and with a low level of functioning at a 12-month follow-up.

**Meaning:**

This study found that, in people with high risk for developing psychosis, functional outcomes are associated with the degree to which their emotion processing is altered.

## Teaser

This case-control study analyzes emotion recognition and neuroimaging data as well as clinical and functional outcomes for individuals at risk for transition to psychosis and those without psychiatric or neurological disorders.

## Introduction

Psychotic disorders are associated with socioemotional dysfunction, which manifests as emotion perception and expression deficits and heightened emotional responsivity^1^ and represents a relatively poor prognosis.^2,3,4^ Neuroimaging studies in established schizophrenia indicate that socioemotional dysfunction is associated with functional^5,6,7,8,9,10,11,12,13^ and structural^14,15,16^ alterations within a corticolimbic circuit that includes the medial prefrontal cortex (MPFC), amygdala, hippocampus, and insula, consistent with postmortem evidence implicating these regions in the disorder.^17^ Preclinical studies in animal models of psychosis suggest that targeting corticolimbic dysregulation during the premorbid phase may prevent the emergence of schizophrenia-like features in adulthood.^18,19^ Human participants at clinical high risk (CHR) for psychosis also show deficits in emotion processing^20,21,22^ and hyperactivation within corticolimbic regions during emotional tasks^23,24,25^ that are qualitatively similar to those seen in patients with schizophrenia. However, the association between alterations in brain regions subserving emotion processing and clinical outcomes in individuals at CHR for psychosis remains unclear.

The primary aim of the present study was to examine the association between emotion processing, gray matter volume (GMV) in brain areas implicated in emotion processing, and clinical and functional outcomes in individuals at CHR for psychosis. We assessed emotion recognition and regional GMVs in individuals at CHR and healthy controls, and we evaluated clinical and functional outcomes in the CHR sample after 12 months of follow-up. We tested the hypothesis that in individuals at CHR, adverse clinical outcomes (the subsequent onset of psychosis or a poor level of functioning) would be associated with abnormalities in emotion recognition (happy, angry, fearful, and neutral faces) and decreased GMV in corticolimbic areas (MPFC, amygdala, hippocampus, and insula) at baseline.^15,26^

## Methods

### Participants

In this case-control study, baseline neuroimaging and emotion processing data and clinical and functional outcomes were collected from 9 psychosis early detection centers (Amsterdam, the Netherlands; Basel, Switzerland; Cologne, Germany; Copenhagen, Denmark; London, United Kingdom; Melbourne, Australia; Paris, France; The Hague, the Netherlands; and Vienna, Austria) from July 1, 2010, to August 31, 2016, as part of the European Network of National Schizophrenia Networks Studying Gene-Environment Interactions (EU-GEI) project.^27^ The present study included the subset of individuals from the total EU-GEI sample (345 help-seeking individuals at CHR and 66 healthy controls) for whom both neuroimaging and emotional processing data were available; eTable 1 in the Supplement shows basic characteristics of individuals in and out of the study). Ethical approval for this study was obtained from the local research ethics committees at each of the 9 sites. All participants provided written informed consent.

### Inclusion and Exclusion Criteria and Baseline Measures

Whether participants met CHR criteria was assessed with the Comprehensive Assessment of At-Risk Mental States (CAARMS).^28^ Exclusion criteria were past or present diagnosis of psychotic disorders, past or present neurological disorder, substance abuse or dependence according to *DSM-IV* criteria, contraindication to scanning, or estimated IQ lower than 60. Healthy controls could not meet the criteria for CHR or have a reported personal or (first-degree) family history of a psychiatric or neurological disorder. Individuals who met CHR criteria and were being treated with antipsychotic medication were not excluded as long as this medication had not been prescribed for a psychotic episode. Data on age, sex, race/ethnicity, and years of education were obtained from the Medical Research Council Sociodemographic Schedule.^29^ At baseline, trained raters assessed participants using the CAARMS^28^ and the Global Assessment of Functioning (GAF) scale (score range: 0-100, with the highest score indicating superior functioning and no symptoms).^30^ Interrater reliability was assessed with mandatory rating of online CAARMS and GAF training videos (eMethods and eTable 2 in the Supplement). Estimated IQ was identified using the shortened Wechsler Adult Intelligence Scale,^31^ cannabis use (yes or no) was assessed with the modified Cannabis Experiences Questionnaire,^32^ and tobacco (cigarettes per day) and alcohol (drinks per week) use were recorded with the Composite International Diagnostic Interview.^33^

### Emotion Recognition Assessment, MRI, and Clinical Follow-up

A computerized version of the Degraded Facial Affect Recognition (DFAR) Task^34^ was used to assess emotion recognition performance. The task included photographs of 4 different actors (2 men and 2 women) portraying 4 different emotions: angry, happy, fearful, and neutral. The task comprised 64 trials, with 16 presentations of each of the 4 emotion categories, shown at 100% and 75% intensity to increase task difficulty.^35,36^ When a face was displayed on the computer screen, a participant indicated its emotional expression by pressing a button. A participant’s DFAR task accuracy was computed on the basis of the total number of neutral, happy, fearful, and angry emotions correctly recognized; higher DFAR scores indicated better performance. The Benton Facial Recognition Test (BFRT)^37^ was used to control for the possibility that impaired facial affect recognition was secondary to a deficit in general facial recognition.^35,36^ Details on BFRT performance are shown in the eResults, eTable 3, and eFigure 1 in the Supplement.

At baseline, 3-T magnetic resonance imaging (MRI) scans were collected from all participants and preprocessed using voxel-based morphometry^38^ implemented on statistical parametric mapping software (SPM12; GNU General Public License). The eMethods and eFigure 2 in the Supplement provide details on MRI acquisition, quality assessment, and preprocessing. At 12 months, the level of overall functioning was assessed with the GAF scale.^30^ Changes in GAF scores over time were also analyzed (eResults and eFigure 3 in the Supplement). Transition or nontransition to psychosis within a 2-year period after baseline was identified using the CAARMS psychosis threshold criteria (eTable 4 in the Supplement), with diagnosis confirmed by the *Structured Clinical Interview for DSM-IV Axis I Disorders*,^39^ administered by a researcher trained in its use.

### Statistical Analysis

#### Demographic and Clinical Data

Analyses of demographic and clinical data were performed in SPSS, version 25 (IBM Corp). The association of group with these measures was examined using 2-sample, unpaired, 2-tailed *t* tests or χ^2^ tests. Effect sizes are expressed as odds ratios (OR) and considered statistically significant at 2-sided *P* < .05. Data were analyzed from October 1, 2018, to April 24, 2019.

#### DFAR Data and Data Integration

Binary logistic regression examined the associations between DFAR task performance and case-control status at baseline, adjusted for age, sex, IQ, site, and BFRT score.^36^ To find the associations between DFAR performance and clinical outcomes, we dichotomized the CHR sample according to transition vs nontransition to psychosis^40^ and in terms of good (GAF score ≥65) vs poor (GAF score <65) overall functioning at follow-up.^30^ A GAF score of 65 was chosen for consistency with the score in previous neuroimaging studies in CHR.^41,42^ Binary logistic regression analyses were performed (transition vs nontransition; good vs poor functioning) with the same covariates. After preprocessing, segmented, normalized, and smoothed GMV images were analyzed in a group using SPM12 to find the associations with DFAR task performance. Individual DFAR task accuracy values were entered as regressors in separate voxelwise analyses of variance to examine the interactions between group status (healthy controls vs CHR; transition vs nontransition; good vs poor functioning) and DFAR task performance on each emotion category, covarying for age, sex, IQ, scanner, and BFRT score. For the imaging analysis, the variable scanner instead of site was used. Although the DFAR task was administered at each site, participants from Amsterdam and The Hague were scanned in Amsterdam, a site that changed scanners during the EU-GEI project (eTable 5 in the Supplement). The analyses of variance also used proportional scaling of the total intracranial volume to adjust for global effects. An initial height threshold of uncorrected *P* < .001 was used to then apply small volume correction for region-of-interest analyses at a voxelwise height threshold of familywise error (FWE) *P* < .05,^43^ using a prespecified bilateral mask. The mask was derived from the WFU_Pitckatlas toolbox in SPM12 and comprised a network implicated in emotion (MPFC, amygdala, hippocampus, and insula). The MPFC and amygdala were chosen because of their central roles in emotion processing^44,45,46^ and because emotion-processing abnormalities in schizophrenia have been associated with volumetric alterations in these regions.^10,12,15,24,47^ The hippocampus was selected because of its key role in the onset of psychosis in preclinical models^48^ and volumetric decreases in this region in individuals at CHR who transition to psychosis.^49^ The insula was chosen because of its involvement in emotion processing^50,51^ and its role in facial emotion recognition in individuals at CHR.^21^

Potential confounding effects of antipsychotic or antidepressant medication (yes or no), substance use (tobacco, cannabis, or alcohol), or baseline levels of CAARMS anxiety or depression symptom severity on regions showing statistically significant DFAR-GMV interactions were assessed in SPSS (antidepressants, substances, and CAARMS anxiety or depression) or SPM (antipsychotic drugs) (eResults in the Supplement). Because our hypotheses involved the association between DFAR-GMV interactions with clinical outcomes, only participants for whom these data were available were included in the present study. Group differences in GMV are currently under analysis (M. J. Kempton, PhD, unpublished data, 2019). Analysis of DFAR task performance and GMV by site or scanner is shown in eTable 6 in the Supplement. Sensitivity analyses data are reported in the eResults, eTables 7 and 8, and eFigures 4 and 5 in the Supplement.

## Results

### Demographic and Clinical Data

At baseline, the participants who had MRI and DFAR data and therefore were included in the study were 213 help-seeking individuals at CHR for psychosis (105 women [49.3%]; mean [SD] age, 22.9 [4.7] years) and 52 healthy controls (25 women [48.1%]; mean [SD] age, 23.3 [4.0] years) (Table 1). Of the 213 individuals at CHR, 193 (90.6%) were naive to antipsychotic medication, and the remaining 20 (9.4%) were receiving low doses of antipsychotics (<1.5 mg haloperidol chlorpromazine equivalents per day). The CHR and healthy control groups did not differ statistically significantly in age (*t* = 0.596; *P* = .55), sex (χ^2^ = 0.025; *P* = .88), race/ethnicity (χ^2^ = 9.023; *P* = .11), BFRT score (*t* = –0.005; *P* = .99), cigarettes (*t* = –1.913; *P* = .06), alcohol (*t* = –0.304; *P* = .76), or cannabis (χ^2^ = 0.403; *P* = .53) use. However, individuals at CHR had fewer years of education (*t* = 3.639; *P* < .001) and lower IQ (*t* = 5.051; *P* < .001).

**Table 1.** Baseline Demographic, Clinical, and Medication Characteristics of Participants

Measure	HC Group (n = 52)	CHR Group (n = 213)	*P* Value	CHR-NT Group (n = 169)	CHR-T Group (n = 44)	*P* Value	CHR-GO Group (n = 39)	CHR-PO Group (n = 91)	*P* Value
Age, mean (SD), y	23.3 (4.0)	22.9 (4.7)	.55	23.0 (4.7)	22.6 (4.7)	.59	23.5 (4.7)	23.1 (5.0)	.66
Sex, No.									
Male	27	108	.88	83	25	.36	20	50	.70
Female	25	105		86	19		19	41	
Years of education, mean (SD)	16.3 (2.9)	14.6 (3.1)	<.001	14.7 (3.1)	14.3 (3.0)	.53	15.5 (2.8)	15.0 (3.2)	.42
Race/ethnicity (% white), %	65.4	72.8	.11	72.8	72.7	.98	71.8	76.9	.41
CAARMS score, mean (SD)									
Positive	0.7 (1.6)	9.9 (4.2)	<.001	9.8 (4.4)	10.6 (3.6)	.24	9.7 (4.5)	10.3 (4.1)	.46
Negative	0.8 (1.7)	7.2 (3.4)	<.001	7.1 (3.5)	7.3 (3.4)	.72	7.3 (3.5)	7.6 (3.2)	.70
Anxiety	0.6 (1.1)	3.1 (1.6)	<.001	3.1 (1.6)	3.2 (1.6)	.84	3.1 (1.6)	3.2 (1.5)	.86
Depression	0.4 (0.9)	3.4 (1.3)	<.001	3.4 (1.3)	3.5 (1.4)	.62	3.3 (1.1)	3.5 (1.2)	.41
Baseline GAF score, mean (SD)	87.2 (9.1)	54.0 (10.0)	<.001	53.7 (9.6)	55.3 (11.6)	.41	53.8 (8.7)	54.2 (11.1)	.86
Antipsychotic drugs, No.									
Total	52	167	NA	131	36	<.001	31	72	.12
No	52	147		122	25		30	62	
Yes	0	20		9	11		1	10	
Antidepressants, No.									
Total	52	167	NA	131	36	.70	31	72	.23
No	52	102		79	23		22	42	
Yes	0	65		52	13		9	30	
Psychological treatment, No.^a^									
Total	51	193	<.001	153	40	.04	33	85	.77
No	47	117		87	30		20	49	
Yes	4	76		66	10		13	36	
Total intracranial volume, mean (SD), mm^3^	1 493 654.5 (178 968.1)	1 493 871.5 (180 458.9)	.99	1 498 994.0 (178 703.8)	1 474 196.6 (187 846.8)	.42	1543 165.2 (169 397.6)	1 492 014.2 (192 273.4)	.15
BFRT score, mean (SD)	22.3 (2.3)	22.3 (2.2)	.99	22.2 (2.2)	23.1 (1.8)	.01	22.3 (2.1)	22.3 (2.3)	.99

Abbreviations: BFRT, Benton Facial Recognition Test; CAARMS, Comprehensive Assessment of At-Risk Mental States; CHR, clinical high risk; CHR-GO, clinical high risk with good overall functioning (GAF ≥65); CHR-NT, clinical high risk–nontransitioned; CHR-PO, clinical high risk with poor overall functioning (GAF <65); CHR-T, clinical high risk–transitioned; DFAR, Degraded Facial Affect Recognition; GAF, Global Assessment of Functioning scale (score range: 0-100, with the highest score indicating superior functioning and no symptoms); HC, healthy controls; MRI, magnetic resonance imaging; NA, not applicable.

^a^ Psychological treatment included counseling sessions, cognitive behavioral therapy, family therapy, psychoeducation, or other form of therapy.

### Clinical Outcomes and Facial Emotional Processing

After 12 months, 39 of the 130 individuals (30.0%) at CHR reinterviewed with the GAF at follow-up had good overall functioning (CHR-GO), whereas 91 (70.0%) had poor overall functioning (CHR-PO). No significant differences at baseline were observed in any clinical or demographic measures between these subgroups (Table 1). Within the 2 years after baseline, 44 individuals at CHR (20.7%) had developed a first episode of psychosis or transitioned (CHR-T). The mean (SD) time to transition in the CHR-T group was 296.3 (257.6) days. Among individuals at CHR, 169 (79.3%) did not develop psychosis within this period, or nontransitioned (CHR-NT). At baseline, the only significant difference in clinical or demographic measures between the CHR-NT and the CHR-T subgroups was that the CHR-T group had a higher BFRT score (*t* = –2.470; *P* = .01) and included more individuals receiving low doses of antipsychotic medications (χ^2^ = 15.028; *P* < .001) (Table 1). The distribution of individuals in the CHR-NT or CHR-T groups among the CHR-GO and CHR-PO follow-up groups is shown in eFigure 6 in the Supplement.

**Figure 1.**
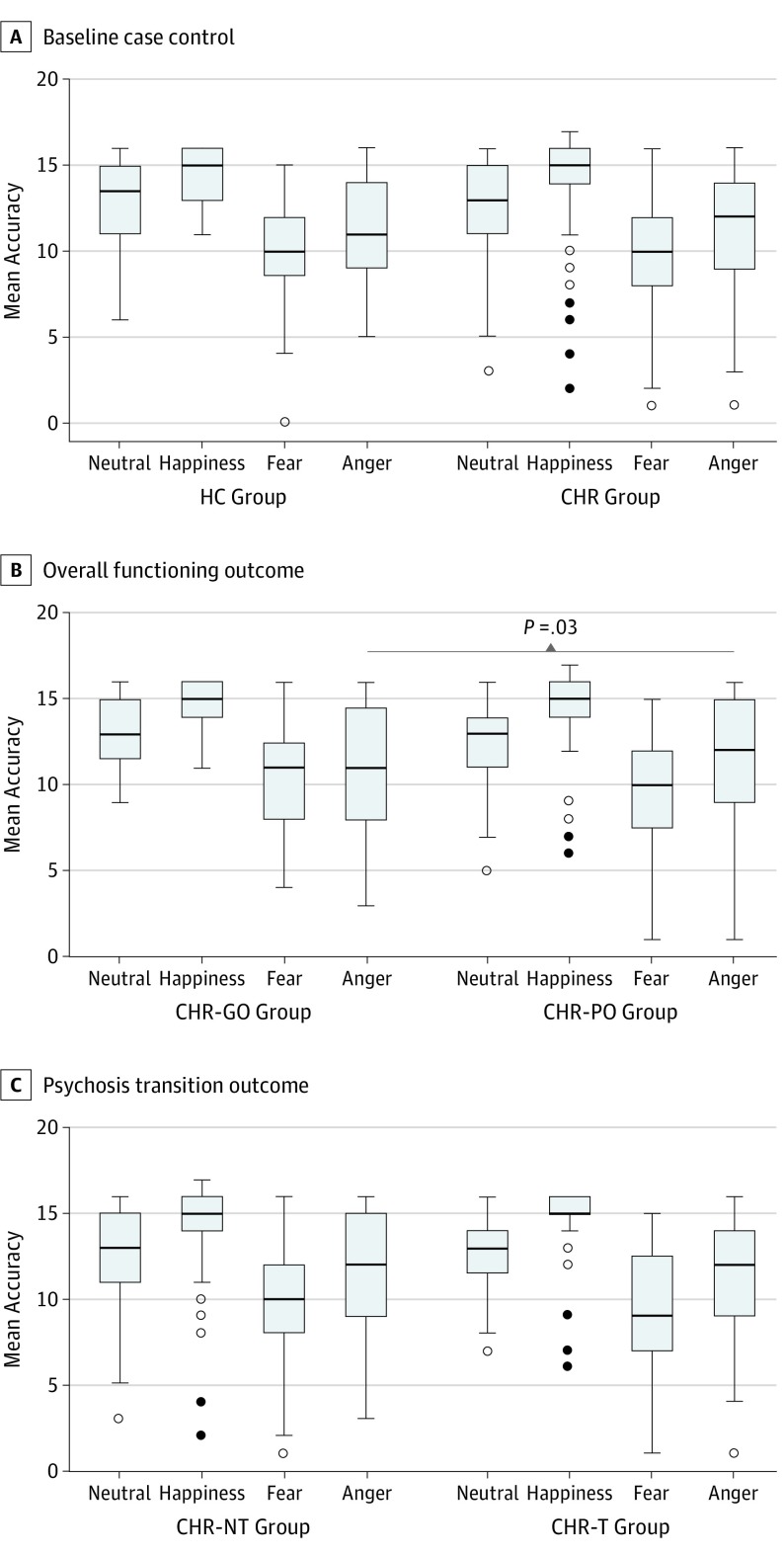
Group Differences in Emotion Recognition A, The healthy control (HC) group comprised 52 participants, and the clinical high risk (CHR) group comprised 213 participants. B, The CHR with good overall functioning (CHR-GO) group comprised 39 participants, and the CHR with poor overall functioning (CHR-PO) group comprised 91 participants. C, The CHR–nontransitioned (CHR-NT) group comprised 169 outcomes, and the CHR–transitioned (CHR-T) group comprised 44 outcomes. The group differences were adjusted for age, sex, IQ, site, and general facial recognition. The horizontal line in each box represents the median; top and bottom box borders, 75th and 25th percentiles, respectively; whiskers, 90th and 10th percentiles; white circles, out values; and black circles, far out values.

At baseline, DFAR task accuracy did not differ for any emotion between the healthy control and the total CHR groups independent of outcomes (Table 2 and Figure 1A). However, within the CHR sample, anger recognition at baseline was significantly associated with the level of functioning at 12-month follow-up; anger recognition was abnormal in the CHR-PO group compared with the CHR-GO group (OR, 0.88; 95% CI, 0.78-0.99; *P* = .03; Table 2 and Figure 1B). No significant associations were observed with CHR-NT and CHR-T outcomes (Table 2 and Figure 1C). See eTable 9 in the Supplement for analysis of potential confounders.

**Table 2.** Group Differences in Facial Emotion Recognition^a^

DFAR Task	HC Group (n = 52) vs CHR Group (n = 213)		CHR-GO Group (n = 39) vs CHR-PO Group (n = 91)		CHR-NT Group (n = 169) vs CHR-T Group (n = 44)	
	OR (95% CI)	*P* Value	OR (95% CI)	*P* Value	OR (95% CI)	*P* Value
Neutral	1.02 (0.86-1.21)	.82	1.03 (0.85-1.24)	.77	0.93 (0.79-1.09)	.37
Happy	1.01 (0.83-1.23)	.91	0.96 (0.76-1.21)	.70	1.03 (0.84-1.25)	.81
Fear	0.89 (0.77-1.02)	.10	1.13 (0.96-1.32)	.13	0.98 (0.85-1.13)	.77
Anger	1.08 (0.96-1.22)	.22	0.88 (0.78-0.99)	.03	1.00 (0.89-1.12)	.96

Abbreviations: CHR, clinical high risk; CHR-GO, clinical high risk with good overall functioning (GAF ≥65); CHR-NT, clinical high risk–nontransitioned; CHR-PO, clinical high risk with poor overall functioning (GAF <65); CHR-T, clinical high risk–transitioned; DFAR, Degraded Facial Affect Recognition; GAF, Global Assessment of Functioning scale; HC, healthy controls; OR, odds ratio.

^a^ Adjusted for age, sex, IQ, site, and general facial recognition.

### DFAR and GMV Data Integration

**Figure 2.**
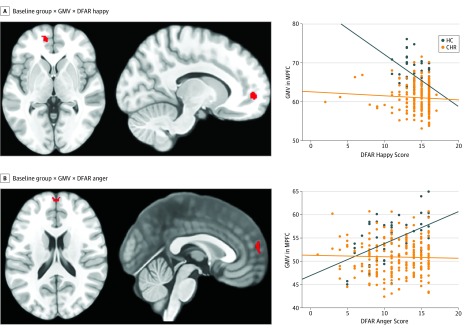
Baseline Associations Between Emotion Recognition (DFAR), Gray Matter Volume (GMV), and Group Status The healthy control (HC; blue) group comprised 52 participants, and the clinical high risk (CHR; orange) group comprised 213 participants. Baseline associations were adjusted for age, sex, IQ, scanner, and general facial recognition (familywise error *P* < .05). MPFC indicates medial prefrontal cortex; DFAR, degraded facial affect recognition. Orange marks are the statistical parametric maps of the interactions between GMV, DFAR performance, and group overlaid on a standard T1 template in MRICron software (NITRC).

At baseline, a significant group × DFAR happy × GMV interaction was observed in the left MPFC (x, y, *z* = –12, 54, 0; *z* = 4.01; FWE *P* = .03). In healthy controls, a negative correlation between the recognition of happiness and MPFC volume was found, which was absent in the CHR sample (Figure 2A). Similarly, a significant group × GMV interaction in the MPFC for DFAR anger was observed (x, y, z = 0, 60, 18; *z* = 3.83; FWE *P* = .049), reflecting a positive correlation between the recognition of anger and GMV in the MPFC among healthy controls that was absent in individuals at CHR (Figure 2B). No other significant interactions with neutral or fearful emotion were found.

#### Functional Outcome and Transition to Psychosis

**Figure 3.**
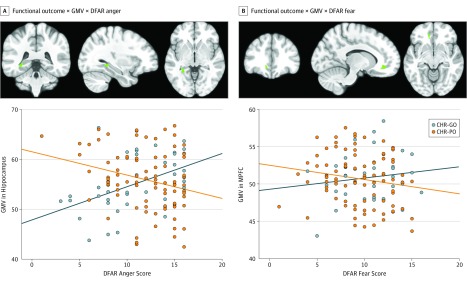
Associations Between 12-Month Functional Outcomes, Gray Matter Volume (GMV), and Degraded Facial Affect Recognition (DFAR) The clinical high risk with good overall functioning (CHR-GO; blue) group comprised 39 participants, and the clinical high risk with poor overall functioning (CHR-PO; orange) group comprised 91 participants. Associations adjusted for age, sex, IQ, scanner, and general facial recognition (familywise error *P* < .05). Blue marks are the statistical parametric maps of the interactions between GMV, DFAR performance, and group overlaid on a standard T1 template in MRICron software (NITRC).

Subdivision of the CHR sample according to level of functioning at follow-up revealed that participants in the CHR-GO group showed a positive association between anger recognition and left hippocampal volume (x, y, z = −32, −40, −3; *z* = 3.91; FWE *P* = .02) and between fear recognition and left MPFC volume (x, y, z = −12, 38, –9; *z* = 3.60; FWE *P* = .02), compared with participants in the CHR-PO group (Figure 3). No other significant group interactions with neutral or happy emotion were observed. No significant group × DFAR × GMV interactions based on transition vs nontransition outcomes were found. Analysis of the potential confounders on all DFAR × GMV interaction data (at baseline and follow-up) rendered the results largely unchanged (eResults; eTable 10; and eFigures 7, 8, and 9 in the Supplement).

The onset of psychosis was not associated with baseline emotion recognition performance (neutral OR, 0.93; 95% CI, 0.79-1.09; *P* = .37; happy OR, 1.03; 95% CI, 0.84-1.25; *P* = .81; fear OR, 0.98; 95% CI, 0.85-1.13; *P* = .77; anger OR, 1.00; 95% CI, 0.89-1.12; *P* = .96). No difference was observed in the association between performance and regional GMVs in individuals at CHR who developed or did not develop psychosis (FWE *P* < .05).

## Discussion

To our knowledge, this case-control study is the first to assess the association between alterations in emotion processing and clinical and functional outcomes in individuals at CHR for psychosis. We studied a large sample of participants at CHR, most of whom were not taking antipsychotic drugs. The main finding was that, in individuals at CHR, a poor functional outcome was associated with baseline abnormalities in both the recognition of angry emotion and in GMV in brain regions implicated in the processing of anger and fear. More specifically, compared with individuals at CHR with poor overall functioning, individuals at CHR with a good functional outcome showed a statistically significant positive association between anger recognition and hippocampal volume as well as between fear recognition and MPFC volume. No associations were found between alterations in emotional processing or in brain regions implicated in emotional processing and the subsequent onset of psychosis in individuals at CHR. However, in healthy controls at baseline, emotion recognition (eg, happy or angry) was significantly associated with MPFC volume, and these associations were absent in the CHR sample. These findings were observed after adjusting for age, sex, scanner, IQ, and BFRT score and remained largely unchanged after removing participants at CHR taking low doses of antipsychotic medications, as well as when examining potential effects of antidepressants or substance use. Overall, these findings suggest that emotional dysfunction and related brain regions may play a role in the development of adverse functional outcomes in individuals at increased risk for psychosis.

Consistent with the main hypothesis is the finding that, in individuals at CHR, a low level of overall functioning at follow-up was associated with baseline alterations in the recognition of angry emotion and decreased anger- and fear-related hippocampal and MPFC volume. Aberrant emotion recognition is a robust factor in poor social^52^ and functional^53^ outcomes in patients with schizophrenia. The hippocampus plays a key role in the pathophysiological process of schizophrenia,^54^ but the clinical and functional importance of changes in this region is still unclear.^55^ Abnormal hippocampal volume has been associated with lower global functioning in both schizophrenia^56,57^ and first-episode psychosis.^58^ In CHR samples, poor functional outcomes have been associated with increased hippocampal glutamate levels,^42^ increased resting hippocampal perfusion,^59^ and altered hippocampal activation.^41^ Decreased MPFC volume has been associated with altered emotional processing in patients with schizophrenia compared with healthy controls.^15,16,26^ In terms of specific emotions, smaller prefrontal volumes have been associated with worse recognition of angry emotion in a large sample of patients with schizophrenia, a finding consistent with ours in the CHR-PO group, which found anger as the only DFAR task impairment in patients after adjusting for age, sex, estimated IQ, and BFRT score.^26^ Furthermore, a recent study using machine learning in individuals at CHR found that baseline MFPC and temporo-parieto-occipital volume reductions were factors in socio-occupational impairments at follow-up.^60^ By linking abnormalities in emotion recognition and emotion-related brain regions to adverse functional outcomes in individuals at CHR, the present study provides further support for the notion that the pathophysiological process of CHR states for psychosis involves emotion-related regions.^14^ These findings may inform the development of new treatments for individuals at CHR, most of whom have deficits in social and role functioning that persist after first presentation.^61,62^

The subsequent onset of psychosis was not associated with abnormalities in either emotion recognition or emotion-related brain regions. This finding was unexpected given that impaired facial emotion recognition has been reported in patients with established schizophrenia^63,64^ and first-episode psychosis,^35,65,66^ and a previous study in at-risk youths reported that lower baseline emotion recognition was associated with psychosis transition.^67^ Nevertheless, these findings are in line with those from another large study of individuals at CHR, which found no association between baseline emotion recognition and the subsequent onset of psychosis,^68^ and with recent meta-analytic evidence that social cognitive deficits are not associated with the subsequent transition to psychosis.^69^ Divergent results may reflect differences in the mean age of the participants and in sample size of the respective studies (23 years in the present study [n = 213], 16 years in Allott et al^67^ [n = 37], and 20 years in Addington et al^68^ [n = 172]). Another potential factor is limited statistical power. Even with relatively large CHR samples, the size of the CHR-T subgroup may be small because only some participants will develop psychosis. This small size may be less of a problem when outcome is defined by level of functioning, as the numbers of participants in subgroups with good and poor functional outcomes may be more similar. Results of the present study suggest that altered emotion processing may predispose individuals at CHR to poor functional outcomes through interactions with prefronto-hippocampal anatomy.

Another finding was that the DFAR-GMV analysis revealed significant differences between the healthy control group and the total CHR group at baseline independent of outcomes. Within healthy controls, MPFC volume was negatively associated with happy emotion recognition, whereas anger recognition was positively associated with MPFC volume (and insula volume after removing participants taking antipsychotic medications); these associations were absent in the CHR sample. Broadly, these findings align with reports that GMV in a topographically similar MPFC region was associated with deficits in social cognitive and emotional tasks in patients with established schizophrenia.^15,16,26^ This ventral portion of the MPFC is involved in monitoring internal affective states and regulates the influence of those states on behavior.^44^ The divergent directionality of the observed DFAR-GMV correlations in healthy controls (negative for happiness, and positive for anger) is interesting and merits further investigation. It may relate to different requirements for prefrontal involvement as a function of emotional valence and its associated emotion regulation requirement or goal.^70^ Because no further significant associations were observed with this a priori region-of-interest analysis or in the complementary whole-brain analysis, these results indicate that the prominent role of the MPFC in emotion recognition may be compromised in the CHR state.

Contrary to our expectations, we found no significant associations between amygdala volumes and DFAR performance between the baseline groups or in association with clinical or functional outcomes. The amygdala plays a key role in emotional processing,^46^ and evidence for abnormal amygdala reactivity has been provided by several functional MRI studies that used emotion processing tasks in patients with schizophrenia.^10,11^ However, because the altered amygdala response in schizophrenia is primarily evident during implicit emotion paradigms,^12^ the lack of association in this study may be attributed to the use of an explicit test (DFAR task) and may align with previous negative findings with the DFAR task by being associated with amygdala volumes in a large sample of patients with schizophrenia.^26^ Future studies examining implicit and explicit emotion processing in CHR samples are needed to expand on this possibility.

### Limitations

The present study has limitations. It assessed functional outcomes in the CHR sample at 12 months and transition or nontransition outcomes within 2 years from baseline. Although most transitions to psychosis seem to occur in this period,^71^ a median duration of the prodromal phase of 64 months has been reported^72^; during a longer follow-up period, additional transitions may have been detected, which could have altered the results. Although a 20% transition rate provides reasonable power, and transition or nontransition outcomes were recorded for all participants at CHR at follow-up, the sample size may still be limited to detect an effect associated with transition. The GAF scale was used to index global functioning, which takes into account current symptom severity and level of functioning.^73^ Nevertheless, complementary analyses of functional outcomes adjusted for psychosis transition outcomes left the results of DFAR task performance and DFAR-GMV associations unchanged, suggesting that GAF scores captured a unique component of outcome beyond psychosis alone. Although combining multicenter data sets increases sensitivity, the application of voxel-based morphometry to large-scale investigations pooling neuroimaging data across sites has some potential limitations. We used the scanner as a covariate to mitigate against the introduction of between-center sources of variability to the data related to, for example, imaging hardware, because of evidence that this approach can suppress scanner effects even when the ratio of cases to controls was unbalanced across sites.^74^ However, recently developed methods such as ComBat (a popular method in genomics for combatting batch effects when combining batches of gene expression microarray data) appear to be successful at harmonizing cortical thickness measurements obtained from multiple sites^75^ and should be considered in future large-scale collaborative imaging studies.

## Conclusions

This case-control study found that poor functional outcome in individuals at CHR of psychosis was associated with baseline abnormalities in the recognition of angry emotion and with abnormal associations between anger and fear emotion recognition and between hippocampal and MPFC volumes. These findings have potential implications for the stratification of individuals at CHR according to subsequent outcomes and suggest that functional outcomes might be improved by interventions that target socioemotional processing.

## Notes

Supplement. **eMethods.** MRI Data Acquisition and Preprocessing; Image Quality Assessment; Assessment of Inter-Rater Reliability (IRR) **eResults.** BFRT Results; Global Functioning Change Over Time; Sensitivity Analyses; Potential Confounders; Analysis of Normality for DFAR-GMV Interaction Data **eTable 1.** Basic Characteristics of EU-GEI Participants In (With DFAR and MRI) and Out (No DFAR or MRI) of the Present Study **eTable 2.** Inter-rater Reliability Analysis of GAF and CAARMS Scores **eTable 3.** Group Differences in Facial Emotion Recognition Removing Participant with Low BFRT Score (Adjusted for Age, Sex, IQ, Site and General Facial Recognition) **eTable 4.** CAARMS Psychosis Threshold Criteria **eTable 5.** Number of Participants by Group in the Different Samples Across Scanners That Had MRI and DFAR Data **eTable 6.** DFAR Performance by Site and GMV by Scanner **eTable 7.** Group Differences in Facial Emotion Recognition (Adjusted for Age, Sex, IQ, Site and General Facial Recognition) Excluding the Sites That Did Not Contribute HC Data **eTable 8.** Group Differences in Facial Emotion Recognition (Adjusted for Age, Sex, IQ, Site and General Facial Recognition) Excluding the Site Contributing One Participant to the CHR-GO vs CHR-PO Analysis **eTable 9.** Group Differences in Facial Emotion Recognition Associated with Longitudinal Outcomes Adjusted for Age, Sex, IQ, Site and General Facial Recognition and Baseline Levels of Prodromal Symptoms (CAARMS Positive) or Global Functioning (GAF) **eTable 10.** Analysis of Potential Confounders on GMV-DFAR Interactions Observed With Baseline Status (HC, CHR) and Functional Outcomes (CHR-GO, CHR-PO) **eFigure 1**. Group x GMV x DFAR Interactions After Removing One Participant With a Low BFRT Score (Covarying for Age, Sex, Scanner, IQ and BRFT) (p_FWE_<0.05) **eFigure 2.** QA Results from the Application of CAT12 to the Structural MRI Images of the 265 Study Participants **eFigure 3. **Partial Correlation Plots of the Associations Between Baseline DFAR Performance and GAF Change Scores (Adjusted for Age, Sex, IQ, Site and BFRT Score) **eFigure 4. **Group x GMV x DFAR Interactions After Excluding the Sites That Did Not Contribute HC Data (Covarying for Age, Sex, Scanner, IQ and BRFT) (p_FWE_<0.05) **eFigure 5. **Group x GMV x DFAR Interactions After Removing the Site Contributing One Participant to the CHR-GO vs CHR-PO Analysis (Covarying for Age, Sex, Scanner, IQ and BRFT) (p_FWE_<0.05) **eFigure 6. **Analysis of the Distribution of CHR-NT/CHR-T Individuals in the CHR-GO/CHR-PO Follow-up Groups **eFigure 7. **Baseline Status x GMV x DFAR Interactions After Removing CHR Participants (n = 20) Treated With Antipsychotics (Covarying for Age, Sex, Scanner, IQ and BRFT) (p_FWE_<0.05) **eFigure 8. **Functional Outcome x GMV x DFAR Interactions After Removing CHR Participants (n = 11) Treated With Antipsychotics (Covarying for Age, Sex, Scanner, IQ and BRFT) (p_FWE_<0.05) **eFigure 9. **Functional Outcome x GMV x DFAR Interactions Covarying for Age, Sex, Scanner, IQ, BRFT and Baseline GAF Scores (p_FWE_<0.05)

## References

[1] Kring AM, Caponigro JM Emotion in schizophrenia: where feeling meets thinking. Curr Dir Psychol Sci. 2010;19(4):255-259. doi:10.1177/0963721410377599 22557707 PMC3340922

[2] Häfner H, Maurer K, Löffler W, an der Heiden W, Hambrecht M, Schultze-Lutter F Modeling the early course of schizophrenia. Schizophr Bull. 2003;29(2):325-340. doi:10.1093/oxfordjournals.schbul.a007008 14552507

[3] Tso IF, Grove TB, Taylor SF Emotional experience predicts social adjustment independent of neurocognition and social cognition in schizophrenia. Schizophr Res. 2010;122(1-3):156-163. doi:10.1016/j.schres.2009.12.007 20051314 PMC2891306

[4] Nikolaides A, Miess S, Auvera I, Müller R, Klosterkötter J, Ruhrmann S Restricted attention to social cues in schizophrenia patients. Eur Arch Psychiatry Clin Neurosci. 2016;266(7):649-661. doi:10.1007/s00406-016-0705-6 27305925

[5] Holt DJ, Weiss AP, Rauch SL, Sustained activation of the hippocampus in response to fearful faces in schizophrenia. Biol Psychiatry. 2005;57(9):1011-1019. doi:10.1016/j.biopsych.2005.01.033 15860342

[6] Holt DJ, Kunkel L, Weiss AP, Increased medial temporal lobe activation during the passive viewing of emotional and neutral facial expressions in schizophrenia. Schizophr Res. 2006;82(2-3):153-162. doi:10.1016/j.schres.2005.09.021 16377154

[7] Hall J, Whalley HC, McKirdy JW, Overactivation of fear systems to neutral faces in schizophrenia. Biol Psychiatry. 2008;64(1):70-73. doi:10.1016/j.biopsych.2007.12.014 18295746

[8] Surguladze S, Russell T, Kucharska-Pietura K, A reversal of the normal pattern of parahippocampal response to neutral and fearful faces is associated with reality distortion in schizophrenia. Biol Psychiatry. 2006;60(5):423-431. doi:10.1016/j.biopsych.2005.11.021 16487943

[9] Taylor SF, Phan KL, Britton JC, Liberzon I Neural response to emotional salience in schizophrenia. Neuropsychopharmacology. 2005;30(5):984-995. doi:10.1038/sj.npp.1300679 15689961

[10] Anticevic A, Van Snellenberg JX, Cohen RE, Repovs G, Dowd EC, Barch DM Amygdala recruitment in schizophrenia in response to aversive emotional material: a meta-analysis of neuroimaging studies. Schizophr Bull. 2012;38(3):608-621. doi:10.1093/schbul/sbq131 21123853 PMC3329999

[11] Li HJ, Chan RC, Gong QY, Facial emotion processing in patients with schizophrenia and their non-psychotic siblings: a functional magnetic resonance imaging study. Schizophr Res. 2012;134(2-3):143-150. doi:10.1016/j.schres.2011.10.019 22113155

[12] Taylor SF, Kang J, Brege IS, Tso IF, Hosanagar A, Johnson TD Meta-analysis of functional neuroimaging studies of emotion perception and experience in schizophrenia. Biol Psychiatry. 2012;71(2):136-145. doi:10.1016/j.biopsych.2011.09.007 21993193 PMC3237865

[13] Seiferth NY, Pauly K, Kellermann T, Neuronal correlates of facial emotion discrimination in early onset schizophrenia. Neuropsychopharmacology. 2009;34(2):477-487. doi:10.1038/npp.2008.93 18580874

[14] Aleman A, Kahn RS Strange feelings: do amygdala abnormalities dysregulate the emotional brain in schizophrenia? Prog Neurobiol. 2005;77(5):283-298. doi:10.1016/j.pneurobio.2005.11.005 16352388

[15] Hooker CI, Bruce L, Lincoln SH, Fisher M, Vinogradov S Theory of mind skills are related to gray matter volume in the ventromedial prefrontal cortex in schizophrenia. Biol Psychiatry. 2011;70(12):1169-1178. doi:10.1016/j.biopsych.2011.07.027 21917239 PMC3432316

[16] Yamada M, Hirao K, Namiki C, Social cognition and frontal lobe pathology in schizophrenia: a voxel-based morphometric study. Neuroimage. 2007;35(1):292-298. doi:10.1016/j.neuroimage.2006.10.046 17240165

[17] Benes FM Amygdalocortical circuitry in schizophrenia: from circuits to molecules. Neuropsychopharmacology. 2010;35(1):239-257. doi:10.1038/npp.2009.116 19727065 PMC3055447

[18] Du Y, Grace AA Peripubertal diazepam administration prevents the emergence of dopamine system hyperresponsivity in the MAM developmental disruption model of schizophrenia. Neuropsychopharmacology. 2013;38(10):1881-1888. doi:10.1038/npp.2013.101 23612434 PMC3746684

[19] Du Y, Grace AA Amygdala hyperactivity in MAM model of schizophrenia is normalized by peripubertal diazepam administration. Neuropsychopharmacology. 2016;41(10):2455-2462. doi:10.1038/npp.2016.42 27000940 PMC4987842

[20] Phillips LK, Seidman LJ Emotion processing in persons at risk for schizophrenia. Schizophr Bull. 2008;34(5):888-903. doi:10.1093/schbul/sbn085 18644853 PMC2518637

[21] Kohler CG, Richard JA, Brensinger CM, Facial emotion perception differs in young persons at genetic and clinical high-risk for psychosis. Psychiatry Res. 2014;216(2):206-212. doi:10.1016/j.psychres.2014.01.023 24582775

[22] Barbato M, Liu L, Cadenhead KS, Theory of mind, emotion recognition and social perception in individuals at clinical high risk for psychosis: findings from the NAPLS-2 cohort. Schizophr Res Cogn. 2015;2(3):133-139. doi:10.1016/j.scog.2015.04.004 27695675 PMC5041592

[23] Seiferth NY, Pauly K, Habel U, Increased neural response related to neutral faces in individuals at risk for psychosis. Neuroimage. 2008;40(1):289-297. doi:10.1016/j.neuroimage.2007.11.020 18187342

[24] Modinos G, Tseng HH, Falkenberg I, Samson C, McGuire P, Allen P Neural correlates of aberrant emotional salience predict psychotic symptoms and global functioning in high-risk and first-episode psychosis. Soc Cogn Affect Neurosci. 2015;10(10):1429-1436. doi:10.1093/scan/nsv035 25809400 PMC4590543

[25] Lee SY, Bang M, Kim KR, Impaired facial emotion recognition in individuals at ultra-high risk for psychosis and with first-episode schizophrenia, and their associations with neurocognitive deficits and self-reported schizotypy. Schizophr Res. 2015;165(1):60-65. doi:10.1016/j.schres.2015.03.026 25864951

[26] Maat A, van Haren NEM, Bartholomeusz CF, Kahn RS, Cahn W Emotion recognition and theory of mind are related to gray matter volume of the prefrontal cortex in schizophrenia. Eur Neuropsychopharmacol. 2016;26(2):255-264. doi:10.1016/j.euroneuro.2015.12.013 26711688

[27] van Os J, Rutten BP, Myin-Germeys I, ; European Network of National Networks studying Gene-Environment Interactions in Schizophrenia (EU-GEI) Identifying gene-environment interactions in schizophrenia: contemporary challenges for integrated, large-scale investigations. Schizophr Bull. 2014;40(4):729-736. doi:10.1093/schbul/sbu069 24860087 PMC4059449

[28] Yung AR, Yuen HP, McGorry PD, Mapping the onset of psychosis: the Comprehensive Assessment of At-Risk Mental States. Aust N Z J Psychiatry. 2005;39(11-12):964-971. doi:10.1080/j.1440-1614.2005.01714.x 16343296

[29] Mallett R Sociodemographic Schedule. London, UK: Section of Social Psychiatry, Institute of Psychiatry; 1997.

[30] Hall RC Global assessment of functioning: a modified scale. Psychosomatics. 1995;36(3):267-275. doi:10.1016/S0033-3182(95)71666-8 7638314

[31] Velthorst E, Levine SZ, Henquet C, To cut a short test even shorter: reliability and validity of a brief assessment of intellectual ability in schizophrenia–a control-case family study. Cogn Neuropsychiatry. 2013;18(6):574-593. doi:10.1080/13546805.2012.731390 23167265

[32] Barkus E, Lewis S Schizotypy and psychosis-like experiences from recreational cannabis in a non-clinical sample. Psychol Med. 2008;38(9):1267-1276. doi:10.1017/S0033291707002619 18205966

[33] Robins LN, Wing J, Wittchen HU, The composite international diagnostic interview: an epidemiologic instrument suitable for use in conjunction with different diagnostic systems and in different cultures. Arch Gen Psychiatry. 1988;45(12):1069-1077. doi:10.1001/archpsyc.1988.01800360017003 2848472

[34] van ’t Wout M, Aleman A, Kessels RP, Larøi F, Kahn RS Emotional processing in a non-clinical psychosis-prone sample. Schizophr Res. 2004;68(2-3):271-281. doi:10.1016/j.schres.2003.09.006 15099609

[35] Catalan A, Gonzalez de Artaza M, Bustamante S, Differences in facial emotion recognition between first episode psychosis, borderline personality disorder and healthy controls. PLoS One. 2016;11(7):e0160056. doi:10.1371/journal.pone.0160056 27467692 PMC4965014

[36] Fett AK, Maat A; GROUP Investigators Social cognitive impairments and psychotic symptoms: what is the nature of their association? Schizophr Bull. 2013;39(1):77-85. doi:10.1093/schbul/sbr058 21697150 PMC3523914

[37] Levin HS. Hamsher KdS, Benton AL A short form of the test of facial recognition for clinical use. J Psychol. 1975;91(2):223-228. doi:10.1080/00223980.1975.9923946 28135466

[38] Ashburner J VBM tutorial. https://www.fil.ion.ucl.ac.uk/~john/misc/VBMclass15.pdf. Published March 12, 2015. Accessed May 8, 2017.

[39] First M, Spitzer R, Gibbon M, Williams JBW Structured Clinical Interview for DSM-IV Axis I Disorders (SCID). New York, NY: New York State Psychiatric Institute Biometrics Research; 1995.

[40] Yung AR, Phillips LJ, McGorry PD, Prediction of psychosis: a step towards indicated prevention of schizophrenia. Br J Psychiatry Suppl. 1998;172(33):14-20. doi:10.1192/S0007125000297602 9764121

[41] Allen P, Chaddock CA, Egerton A, Functional outcome in people at high risk for psychosis predicted by thalamic glutamate levels and prefronto-striatal activation. Schizophr Bull. 2015;41(2):429-439. doi:10.1093/schbul/sbu115 25123110 PMC4332951

[42] Bossong MG, Antoniades M, Azis M, Association of hippocampal glutamate levels with adverse outcomes in individuals at clinical high risk for psychosis. JAMA Psychiatry. 2019;76(2):199-207. doi:10.1001/jamapsychiatry.2018.3252 30427993 PMC6440239

[43] Eklund A, Nichols TE, Knutsson H Cluster failure: why fMRI inferences for spatial extent have inflated false-positive rates. Proc Natl Acad Sci U S A. 2016;113(28):7900-7905. doi:10.1073/pnas.1602413113 27357684 PMC4948312

[44] Amodio DM, Frith CD Meeting of minds: the medial frontal cortex and social cognition. Nat Rev Neurosci. 2006;7(4):268-277. doi:10.1038/nrn1884 16552413

[45] Kober H, Barrett LF, Joseph J, Bliss-Moreau E, Lindquist K, Wager TD Functional grouping and cortical-subcortical interactions in emotion: a meta-analysis of neuroimaging studies. Neuroimage. 2008;42(2):998-1031. doi:10.1016/j.neuroimage.2008.03.059 18579414 PMC2752702

[46] Phelps EA, LeDoux JE Contributions of the amygdala to emotion processing: from animal models to human behavior. Neuron. 2005;48(2):175-187. doi:10.1016/j.neuron.2005.09.025 16242399

[47] Phillips ML, Drevets WC, Rauch SL, Lane R Neurobiology of emotion perception II: implications for major psychiatric disorders. Biol Psychiatry. 2003;54(5):515-528. doi:10.1016/S0006-3223(03)00171-9 12946880

[48] Lieberman JA, Girgis RR, Brucato G, Hippocampal dysfunction in the pathophysiology of schizophrenia: a selective review and hypothesis for early detection and intervention. Mol Psychiatry. 2018;23(8):1764-1772. doi:10.1038/mp.2017.249 29311665 PMC6037569

[49] Mechelli A, Riecher-Rössler A, Meisenzahl EM, Neuroanatomical abnormalities that predate the onset of psychosis: a multicenter study. Arch Gen Psychiatry. 2011;68(5):489-495. doi:10.1001/archgenpsychiatry.2011.42 21536978

[50] Singer T, Critchley HD, Preuschoff K A common role of insula in feelings, empathy and uncertainty. Trends Cogn Sci. 2009;13(8):334-340. doi:10.1016/j.tics.2009.05.001 19643659

[51] Fusar-Poli P, Placentino A, Carletti F, Functional atlas of emotional faces processing: a voxel-based meta-analysis of 105 functional magnetic resonance imaging studies. J Psychiatry Neurosci. 2009;34(6):418-432. 19949718 PMC2783433

[52] Hooker C, Park S Emotion processing and its relationship to social functioning in schizophrenia patients. Psychiatry Res. 2002;112(1):41-50. doi:10.1016/S0165-1781(02)00177-4 12379449

[53] Fett AK, Viechtbauer W, Dominguez MD, Penn DL, van Os J, Krabbendam L The relationship between neurocognition and social cognition with functional outcomes in schizophrenia: a meta-analysis. Neurosci Biobehav Rev. 2011;35(3):573-588. doi:10.1016/j.neubiorev.2010.07.001 20620163

[54] Tamminga CA, Stan AD, Wagner AD The hippocampal formation in schizophrenia. Am J Psychiatry. 2010;167(10):1178-1193. doi:10.1176/appi.ajp.2010.09081187 20810471

[55] Heckers S, Konradi C Hippocampal pathology in schizophrenia. Curr Top Behav Neurosci. 2010;4:529-553. doi:10.1007/7854_2010_43 21312412

[56] Anvari AA, Friedman LA, Greenstein D, Gochman P, Gogtay N, Rapoport JL Hippocampal volume change relates to clinical outcome in childhood-onset schizophrenia. Psychol Med. 2015;45(12):2667-2674. doi:10.1017/S0033291715000677 25936396

[57] Andreasen NC, Nopoulos P, Magnotta V, Pierson R, Ziebell S, Ho BC Progressive brain change in schizophrenia: a prospective longitudinal study of first-episode schizophrenia. Biol Psychiatry. 2011;70(7):672-679. doi:10.1016/j.biopsych.2011.05.017 21784414 PMC3496792

[58] Lappin JM, Morgan C, Chalavi S, Bilateral hippocampal increase following first-episode psychosis is associated with good clinical, functional and cognitive outcomes. Psychol Med. 2014;44(6):1279-1291. doi:10.1017/S0033291713001712 23866084

[59] Allen P, Chaddock CA, Egerton A, Resting hyperperfusion of the hippocampus, midbrain, and basal ganglia in people at high risk for psychosis. Am J Psychiatry. 2016;173(4):392-399. doi:10.1176/appi.ajp.2015.15040485 26684922

[60] Koutsouleris N, Kambeitz-Ilankovic L, Ruhrmann S, ; PRONIA Consortium Prediction models of functional outcomes for individuals in the clinical high-risk state for psychosis or with recent-onset depression: a multimodal, multisite machine learning analysis. JAMA Psychiatry. 2018;75(11):1156-1172. doi:10.1001/jamapsychiatry.2018.2165 30267047 PMC6248111

[61] Lin A, Wood SJ, Nelson B, Neurocognitive predictors of functional outcome two to 13 years after identification as ultra-high risk for psychosis. Schizophr Res. 2011;132(1):1-7. doi:10.1016/j.schres.2011.06.014 21763109

[62] Addington J, Cornblatt BA, Cadenhead KS, At clinical high risk for psychosis: outcome for nonconverters. Am J Psychiatry. 2011;168(8):800-805. doi:10.1176/appi.ajp.2011.10081191 21498462 PMC3150607

[63] Comparelli A, Corigliano V, De Carolis A, Emotion recognition impairment is present early and is stable throughout the course of schizophrenia. Schizophr Res. 2013;143(1):65-69. doi:10.1016/j.schres.2012.11.005 23218561

[64] Chan RC, Li H, Cheung EF, Gong QY Impaired facial emotion perception in schizophrenia: a meta-analysis. Psychiatry Res. 2010;178(2):381-390. doi:10.1016/j.psychres.2009.03.035 20483476

[65] Thompson A, Papas A, Bartholomeusz C, Social cognition in clinical “at risk” for psychosis and first episode psychosis populations. Schizophr Res. 2012;141(2-3):204-209. doi:10.1016/j.schres.2012.08.007 22959742

[66] Edwards J, Pattison PE, Jackson HJ, Wales RJ Facial affect and affective prosody recognition in first-episode schizophrenia. Schizophr Res. 2001;48(2-3):235-253. doi:10.1016/S0920-9964(00)00099-2 11295377

[67] Allott KA, Schäfer MR, Thompson A, Emotion recognition as a predictor of transition to a psychotic disorder in ultra-high risk participants. Schizophr Res. 2014;153(1-3):25-31. doi:10.1016/j.schres.2014.01.037 24552619

[68] Addington J, Piskulic D, Perkins D, Woods SW, Liu L, Penn DL Affect recognition in people at clinical high risk of psychosis. Schizophr Res. 2012;140(1-3):87-92. doi:10.1016/j.schres.2012.06.012 22763425 PMC3460803

[69] van Donkersgoed RJ, Wunderink L, Nieboer R, Aleman A, Pijnenborg GH Social cognition in individuals at ultra-high risk for psychosis: a meta-analysis. PLoS One. 2015;10(10):e0141075. doi:10.1371/journal.pone.0141075 26510175 PMC4624797

[70] Kim SH, Hamann S Neural correlates of positive and negative emotion regulation. J Cogn Neurosci. 2007;19(5):776-798. doi:10.1162/jocn.2007.19.5.776 17488204

[71] Fusar-Poli P, Bonoldi I, Yung AR, Predicting psychosis: meta-analysis of transition outcomes in individuals at high clinical risk. Arch Gen Psychiatry. 2012;69(3):220-229. doi:10.1001/archgenpsychiatry.2011.1472 22393215

[72] Schultze-Lutter F, Rahman J, Ruhrmann S, Duration of unspecific prodromal and clinical high risk states, and early help-seeking in first-admission psychosis patients. Soc Psychiatry Psychiatr Epidemiol. 2015;50(12):1831-1841. doi:10.1007/s00127-015-1093-3 26155901

[73] Nelson B, Yuen HP, Wood SJ, Long-term follow-up of a group at ultra high risk (“prodromal”) for psychosis: the PACE 400 study. JAMA Psychiatry. 2013;70(8):793-802. doi:10.1001/jamapsychiatry.2013.1270 23739772

[74] Takao H, Hayashi N, Ohtomo K Effects of study design in multi-scanner voxel-based morphometry studies. Neuroimage. 2014;84:133-140. doi:10.1016/j.neuroimage.2013.08.046 23994315

[75] Fortin JP, Cullen N, Sheline YI, Harmonization of cortical thickness measurements across scanners and sites. Neuroimage. 2018;167:104-120. doi:10.1016/j.neuroimage.2017.11.024 29155184 PMC5845848

