# Supplementary material for: Association of Adverse Outcomes With Emotion Processing and Its Neural Substrate in Individuals at Clinical High Risk for Psychosis
Source: JAMA Psychiatry. 2019 Nov 13;77(2):190–200. doi: 10.1001/jamapsychiatry.2019.3501 (PMC6865249; doi:10.1001/jamapsychiatry.2019.3501)
Supplement: Supplement. — eMethods. MRI Data Acquisition and Preprocessing; Image Quality Assessment; Assessment of Inter-Rater Reliability (IRR) eResults. BFRT Results; Global Functioning Change Over Time; Sensitivity Analyses; Potential Confounders; Analysis of Normality for DFAR-GMV Interaction Data eTable 1. Basic Characteristics of EU-GEI Participants In (With DFAR and MRI) and Out (No DFAR or MRI) of the Present Study eTable 2. Inter-rater Reliability Analysis of GAF and CAARMS Scores eTable 3. Group Differences in Facial Emotion Recognition Removing Participant with Low BFRT Score (Adjusted for Age, Sex, IQ, Site and General Facial Recognition) eTable 4. CAARMS Psychosis Threshold Criteria eTable 5. Number of Participants by Group in the Different Samples Across Scanners That Had MRI and DFAR Data eTable 6. DFAR Performance by Site and GMV by Scanner eTable 7. Group Differences in Facial Emotion Recognition (Adjusted for Age, Sex, IQ, Site and General Facial Recognition) Excluding the Sites That Did Not Contribute HC Data eTable 8. Group Differences in Facial Emotion Recognition (Adjusted for Age, Sex, IQ, Site and General Facial Recognition) Excluding the Site Contributing One Participant to the CHR-GO vs CHR-PO Analysis eTable 9. Group Differences in Facial Emotion Recognition Associated with Longitudinal Outcomes Adjusted for Age, Sex, IQ, Site and General Facial Recognition and Baseline Levels of Prodromal Symptoms (CAARMS Positive) or Global Functioning (GAF) eTable 10. Analysis of Potential Confounders on GMV-DFAR Interactions Observed With Baseline Status (HC, CHR) and Functional Outcomes (CHR-GO, CHR-PO) eFigure 1. Group x GMV x DFAR Interactions After Removing One Participant With a Low BFRT Score (Covarying for Age, Sex, Scanner, IQ and BRFT) (pFWE<0.05) eFigure 2. QA Results from the Application of CAT12 to the Structural MRI Images of the 265 Study Participants eFigure 3. Partial Correlation Plots of the Associations Between Baseline DFAR Performance and GAF Change Scores [file jamapsychiatry-77-190-s001.pdf]

# Supplementary Online Content

Modinos G, Kempton MJ, Tognin S, et al; EU-GEI High Risk Study Group. Association of adverse outcomes with emotion processing and its neural substrate in individuals at clinical high risk for psychosis. *JAMA Psychiatry.* Published online November 13, 2019. doi: 10.1001/jamapsychiatry.2019.3501

**eMethods.** MRI Data Acquisition and Preprocessing; Image Quality Assessment; Assessment of Inter-Rater Reliability (IRR)
**eResults.** BFRT Results; Global Functioning Change Over Time; Sensitivity Analyses; Potential Confounders; Analysis of Normality for DFAR-GMV Interaction Data
**eTable 1.** Basic Characteristics of EU-GEI Participants In (With DFAR and MRI) and Out (No DFAR or MRI) of the Present Study
**eTable 2.** Inter-rater Reliability Analysis of GAF and CAARMS Scores
**eTable 3.** Group Differences in Facial Emotion Recognition Removing Participant with Low BFRT Score (Adjusted for Age, Sex, IQ, Site and General Facial Recognition)
**eTable 4**. CAARMS Psychosis Threshold Criteria
**eTable 5.** Number of Participants by Group in the Different Samples Across Scanners That Had MRI and DFAR Data
**eTable 6.** DFAR Performance by Site and GMV by Scanner
**eTable 7**. Group Differences in Facial Emotion Recognition (Adjusted for Age, Sex, IQ, Site and General Facial Recognition) Excluding the Sites That Did Not Contribute HC Data
**eTable 8.** Group Differences in Facial Emotion Recognition (Adjusted for Age, Sex, IQ, Site and General Facial Recognition) Excluding the Site Contributing One Participant to the CHR-GO vs CHR-PO Analysis
**eTable 9.** Group Differences in Facial Emotion Recognition Associated with Longitudinal Outcomes Adjusted for Age, Sex, IQ, Site and General Facial Recognition and Baseline Levels of Prodromal Symptoms (CAARMS Positive) or Global Functioning (GAF)
**eTable 10.** Analysis of Potential Confounders on GMV-DFAR Interactions Observed With Baseline Status (HC, CHR) and Functional Outcomes (CHR-GO, CHR-PO)
**eFigure 1.** Group x GMV x DFAR Interactions After Removing One Participant With a Low BFRT Score (Covarying for Age, Sex, Scanner, IQ and BRFT) ($p_{FWE}<0.05$)
**eFigure 2.** QA Results from the Application of CAT12 to the Structural MRI Images of the 265 Study Participants
**eFigure 3.** Partial Correlation Plots of the Associations Between Baseline DFAR Performance and GAF Change Scores (Adjusted for Age, Sex, IQ, Site and BFRT Score)
**eFigure 4**. Group x GMV x DFAR Interactions After Excluding the Sites That Did Not Contribute HC Data (Covarying for Age, Sex, Scanner, IQ and BRFT) ($p_{FWE}<0.05$)

©2019 Modinos G et al. *JAMA Psychiatry*.

**eFigure 5**. Group x GMV x DFAR Interactions After Removing the Site Contributing One Participant to the CHR-GO vs CHR-PO Analysis (Covarying for Age, Sex, Scanner, IQ and BRFT) ($p_{FWE}$<0.05)
**eFigure 6.** Analysis of the Distribution of CHR-NT/CHR-T Individuals in the CHR-GO/CHR-PO Follow-up Groups
**eFigure 7.** Baseline Status x GMV x DFAR Interactions After Removing CHR Participants (n=20) Treated With Antipsychotics (Covarying for Age, Sex, Scanner, IQ and BRFT) ($p_{FWE}$<0.05)
**eFigure 8.** Functional Outcome x GMV x DFAR Interactions After Removing CHR Participants (n=11) Treated With Antipsychotics (Covarying for Age, Sex, Scanner, IQ and BRFT) ($p_{FWE}$<0.05)
**eFigure 9.** Functional Outcome x GMV x DFAR Interactions Covarying for Age, Sex, Scanner, IQ, BRFT and Baseline GAF Scores ($p_{FWE}$<0.05)

This supplementary material has been provided by the authors to give readers additional information about their work.

©2019 Modinos G et al. *JAMA Psychiatry*.

**eMethods.** MRI Data Acquisition and Preprocessing; Image Quality Assessment; Assessment of Inter-Rater Reliability (IRR)

**MRI Data Acquisition and Preprocessing.**

For all participants, a three-dimensional T1-weighted inversion recovery prepared gradient echo sequence was obtained at 3T with voxel size: 1 x 1 x 1.2mm. Participants were recruited at 9 different clinical sites: Amsterdam, Basel, Cologne, Copenhagen, London, Melbourne, Paris, The Hague, Vienna. Amsterdam and The Hague participants were scanned at the same site in Amsterdam; given that this site underwent a scanner change halfway through the project, we modelled "scanner" in all behavioral and imaging analyses instead of "site" (eTable 5).

Structural images were preprocessed using the Voxel-Based Morphometry protocol[1] implemented in SPM12, running on Matlab 9.2 (The MathWorks, USA). The following steps were applied: (1) Segmentation of all images into grey matter (GM), white matter (WM), and cerebrospinal fluid (CSF) partitions; (2) Warping of GM partitions into a new study-specific reference space, to provide increased accuracy of inter-subject alignment; (3) Normalization of the warped GM partitions to the MNI space, to generate smoothed (10mm FWHM), spatially normalized and modulated GM images in Montreal Neuroanatomical Imaging (MNI) space. Total intracranial volume was calculated for each subject by summing together the voxel values of grey matter, white matter and cerebrospinal fluid from the original tissue partitions using the ImCalc function in SPM12.

**Image Quality Assessment.**

Image quality assessment (QA) was performed for all of the structural images. This involved careful visual inspection of all structural images by an experienced neuroimaging researcher (MK), and resulted in the exclusion of 3 participants (1 with large congenital cyst, 1 with distortion from brace, one with movement artefact). We also applied CAT12 to the structural MRI images of the 265 study

participants as additional QA measure (eFigure 2). This analysis revealed two volumes of with a relatively low quality rating; these were carefully checked again and no artefacts were identified.

**Assessment of Inter-Rater Reliability (IRR).**

Krippendorff's α were calculated across the raters of the different EU-GEI sites to generate a measure of IRR. An IRR of > 0.7 was deemed acceptable. In order to become an EU-GEI rater, researchers had to pass the online training course, which entailed rating GAF and CAARMS training videos. After the initial training, new IRR videos appeared online and had to be scored at least once a year. These videos and vignettes had to be rated by all researchers recruiting participants for the EU-GEI project. Analysis of IRR across raters across the EU-GEI sites are shown in eTable 2.

**eResults.** BFRT Results; Global Functioning Change Over Time; Sensitivity Analyses; Potential Confounders; Analysis of Normality for DFAR-GMV Interaction Data

**Benton Facial Recognition Test**

The BFRT short form was used,[2] in which a target face is presented centered above six stimulus faces. In the first six trials, only one of the six stimulus faces is identical to the target face. In the following seven trials, three of the stimulus faces match the target. The test involves a total of 13 trials requiring a total of 27 responses (scoring ranges from 0-27). Scores of 21 or above are interpreted as being well within the normal range, and scores of 16 or below are interpreted as being impaired. Out of the 265 participants included in the study, only one participant scored below 16 (score = 15). BFRT scores were included as covariates of no interest in all analyses, as reported in the manuscript (group differences in DFAR performance, group differences in DFAR-GMV interactions).

Additional analyses removing the participant with a score below 16 from the tests of group differences in DFAR performance revealed that all results remained unchanged (eTable 3). Similarly, additional analyses with this participant removed from the tests of group differences between HC and CHR in DFAR-GMV associations did not change the results (eFigure 1). The DFAR anger x GMV interaction in the MPFC remained significant (xyz=0, 60, 18, Z=3.81; $p_{FWE}$=0.03), as did the group x DFAR happy x GMV interaction in the left MPFC (xyz=-12, 54, 0; Z=3.98; $p_{FWE}$=0.03), and the lack of group effects for interactions with neutral or fearful emotion. Removing this participant from the tests of group differences between CHR-GO and CHR-PO in DFAR-GMV associations did not change the results either: the left hippocampal finding for DFAR anger remained unchanged (xyz=−32, −40, −3; Z=3.79; $p_{FWE}$=0.02), as did the left MPFC association with DFAR fear (xyz=−12, 38, -9; Z=3.69; $p_{FWE}$=0.049). Other group interactions with neutral or happy emotion remained non-significant. Finally, the lack of

significant group x DFAR x GMV interactions based on transition vs nontransition outcomes also remained unchanged after removing the participant with a low BFRT score.

**Analysis of global functioning change over time**

We calculated GAF change over time as the difference between scores at the baseline and follow-up scores ($\Delta GAF = GAF_{follow\text{-}up} - GAF_{baseline}$) for those CHR participants in whom follow-up GAF ratings were available (n=130, as per main manuscript page 12). The mean [SD] ΔGAF was 4.20 [17.02].

**Relationship between change in global functioning over time and main study measures**

*DFAR performance*

Partial correlation was performed in SPSS to examine associations between DFAR performance (angry, happy, fearful, and neutral) and GAF change, adjusted for age, sex, IQ, site and BFRT score. This analysis revealed a significant positive correlation (Bonferroni-corrected at p=0.05/4=0.01) between DFAR fear and GAF change (r=0.309, p=0.001), indicating that the better recognition of fear at baseline, the greater the improvement in the GAF score over the follow-up period. There were no other significant correlations (DFAR neutral: r=0.061, p=0.52; DFAR happy: r=0.010, p=0.92; DFAR angry: r=0.073, p=0.44) (eFigure 3).

*DFAR-GMV associations*

Complementary analyses tested whether regions showing between-group differences in the CHR sample versus healthy controls analysis (MPFC – DFAR happy and MPFC – DFAR Anger) were related to longitudinal changes in GAF score. Individual values from the significant clusters in were extracted from SPM and Pearson's product-moment correlation analyses were performed in SPSS with GAF change scores. This analysis revealed no significant associations with GAF change for either MPFC – DFAR happy (r=0.010, p=0.91) or MPFC – DFAR anger (r=0.103, p=0.24).

**Sensitivity analyses for possible site/scanner effects on DFAR performance and DFAR-GMV associations**

***Baseline Status***

*DFAR performance*

Re-analysis of DFAR performance excluding the sites that did not contribute HC participants did not change the results, which remained non-significant (eTable 7).

*Integration of DFAR and GMV data*

Removing the sites that did not contribute HC data from the DFAR-GMV analysis did also not change the results. With a total of n = 52 HC vs n = 115 CHR, the group interaction for DFAR Happy x left MPFC GMV remained unchanged (xyz=−10, 54, 0; Z=3.71; $p_{FWE}$=0.045), as did the group interaction for DFAR Anger x MPFC GMV (xyz=0, 60, 16; Z=3.91; $p_{FWE}$=0.04) (eFigure 4).

***Functional Outcome***

*DFAR performance*

Re-analysis of DFAR performance excluding the site that contributed only one participant to the CHR-GO vs CHR-PO analysis did not change the results: anger recognition at baseline was significantly associated with the level of functioning at 12 months follow-up (p=0.03; eTable 8).

*Integration of DFAR and GMV data*

After removing the site that only contributed one subject to the CHR-GO vs CHR-PO analysis, the left hippocampal finding for DFAR anger remained unchanged (xyz=−32, −40, −3; Z=3.89; pFWE=0.02), but the left MPFC finding (DFAR fear) dropped below the significance threshold (xyz=−12, 38, -9; Z=3.59; pFWE=0.06) (eFigure 5).

**Analysis of potential confounders**

*Facial Emotional Processing*

The functional outcome results remained unchanged after adjusting for baseline prodromal symptoms, baseline GAF scores, or transition/nontransition outcomes (eTable 9).

*Integration of DFAR and GMV data*

**Baseline**. The results were not significantly associated with substance use or levels of anxiety/depressive symptoms (eTable 10), and were unchanged after excluding the minority of CHR participants (N=20) who were taking antipsychotics (Happy: left MPFC, xyz=−12, −54, 0; Z=4.01; $p_{FWE}$=0.03; Anger: MPFC, xyz=0, 60, 16; Z=3.87; $p_{FWE}$=0.04; eFigure 7). Antidepressant use influenced the anger-related MPFC finding, which was stronger in CHR individuals who were not taking antidepressants compared to those who were ($F_{1,165}$=8.225, p=0.005) (eTable 10).

**Functional outcome.** The results were not associated with substances or antidepressant use, or with levels of anxiety/depressive symptoms (eTable 10). After removing participants on antipsychotics (CHR-GO: 1, CHR-PO: 10), the left hippocampal finding for DFAR anger remained unchanged (xyz=−32, −40, −3; Z=3.42; $p_{FWE}$=0.04), but the left MPFC finding (DFAR fear) was no longer significant. However, a new significant effect emerged in the left insula, with CHR-GO showing a stronger (positive) association compared to CHR-PO (xyz=−39, 16, −12; Z=3.64; $p_{FWE}$=0.03) (eFigure 8). Complementary analyses including baseline GAF scores (eFigure 9) did not change the results. These results also remained unchanged after correcting for transition outcomes (anger and left hippocampus: xyz=−32, −40, −3; Z=3.85; $p_{FWE}$=0.02; fear and left MPFC: xyz=−12, 38, -9; Z=3.46; $p_{FWE}$=0.02).

**Transition to psychosis**. Results did not change after removing CHR participants on antipsychotics or adjusting for baseline prodromal symptom scores.

**Analysis of normality for DFAR-GMV interaction data**

©2019 Modinos G et al. *JAMA Psychiatry*.

We examined whether normality could be assumed for the original DFAR-GMV differences analysis. We extracted the individual GMV values from the regions showing group differences between HCs and CHRs (MPFC-DFAR happy and MPFC-DFAR angry) from the corresponding SPM analysis using MarsBaR[3], then applied Shapiro-Wilk's W test in SPSS to determine whether the underlying distribution was normal. This confirmed that the data are normally distributed:

- MPFC-DFAR happy
  - HC (n=52): Shapiro-Wilk's W = 0.984, p = 0.72
  - CHR (n=213): Shapiro-Wilk's W = 0.990, p = 0.16
- MPFC-DFAR angry
  - HC (n=52): Shapiro-Wilk's W = 0.985, p = 0.74
  - CHR (n=213): Shapiro-Wilk's W = 0.992, p = 0.27

©2019 Modinos G et al. *JAMA Psychiatry*.

**eTable 1.** Basic Characteristics of EU-GEI Participants In (With DFAR and MRI) and Out (No DFAR or MRI) of the Present Study

| **Measure** | **HC-in N=52** | **HC-out N=14** | **P** | **CHR-in N=213** | **CHR-out N=89*** | **P** |
|---|---|---|---|---|---|---|
| Age (years) | 23.3 (4.0) | 21.4 (4.3) | 0.12 | 22.9 (4.7) | 22.5 (5.4) | 0.46 |
| Gender (male/female) | 27/25 | 7/7 | 0.90 | 108/105 | 46/43 | 0.88 |
| Years of education | 16.3 (2.9) | 15.4 (2.3) | 0.29 | 14.6 (3.1) | 14.2 (2.8) | 0.30 |
| Ethnicity (% white) | **65.4%** | **57.1%** | **0.01** | **73.1%** | **66.3%** | **0.02** |
| CAARMS Positive score | 0.7 (1.6) | 1.77 (2.9) | 0.24 | 9.9 (4.2) | 10.4 (3.5) | 0.43 |
| CAARMS Negative score | **0.8 (1.7)** | **3.4 (3.7)** | **0.02** | 7.2 (3.4) | 6.5 (3.7) | 0.12 |
| CAARMS Anxiety score | 0.6 (1.1) | 0.9 (1.5) | 0.45 | 3.1 (1.6) | 3.0 (1.6) | 0.59 |
| CAARMS Depression score | **0.4 (0.9)** | **1.8 (2.0)** | **0.02** | 3.4 (1.3) | 3.3 (1.4) | 0.48 |
| Baseline GAF score | **87.2 (9.1)** | **79.4 (8.0)** | **0.01** | 53.9 (10.0) | 55.2 (9.9) | 0.32 |

CHR-in, clinical high risk included in the study; CHR-out, clinical high risk not included in the study, HC-in, healthy controls included in the study**;** HC-out, healthy controls not included in the study, CAARMS, community assessment of at-risk mental states, GAF, global assessment of functioning.
* Two sites within the EU-GEI network did not have access to an MRI scanner; CHR individuals from those sites (n = 43) are excluded from this analysis.

©2019 Modinos G et al. *JAMA Psychiatry*.

**eTable 2.** Inter-rater Reliability Analysis of GAF and CAARMS Scores

| **Assessment Scale** | **Reliability Measurement** |
|---|---|
| GAF | 0.83 |
| CAARMS (Positive items, Intensity scores) | 0.78 |
| CAARMS (Positive items, Frequency scores) | 0.90 |

©2019 Modinos G et al. *JAMA Psychiatry*.

**eTable 3.** Group Differences in Facial Emotion Recognition Removing Participant with Low BFRT Score (Adjusted for Age, Sex, IQ, Site and General Facial Recognition)

| **DFAR** | **HC (N=52) vs CHR (N=212)** | | | **CHR-GO (N=39) vs CHR-PO (N=91)** | | | **CHR-NT (N=169) vs CHR-T (N=44)** | | |
|---|---|---|---|---|---|---|---|---|---|
| | OR | P | 95% CI | OR | P | 95% CI | OR | P | 95% CI |
| Neutral | 1.01 | 0.90 | 0.85-1.20 | 1.02 | 0.87 | 0.84-1.23 | 0.93 | 0.39 | 0.79-1.10 |
| Happy | 0.99 | 0.99 | 0.82-1.22 | 0.94 | 0.62 | 0.74-1.19 | 1.03 | 0.78 | 0.84-1.26 |
| Fear | 0.87 | 0.06 | 0.75-1.00 | 1.12 | 0.18 | 0.95-1.31 | 0.98 | 0.82 | 0.85-1.14 |
| Anger | 1.01 | 0.13 | 0.97-1.24 | **0.88** | **0.04** | **0.78-0.99** | 1.00 | 0.89 | 0.89-1.11 |

CHR: clinical high risk, CHR-GO: clinical high risk good outcome (GAF≥65), CHR-NT: clinical high risk nontransition, CHR-PO: clinical high risk poor outcome (GAF<65), CHR-T: clinical high risk transition, CI, confidence intervals, HC: healthy controls, OR: odds ratio.

©2019 Modinos G et al. *JAMA Psychiatry*.

**eTable 4**. CAARMS Psychosis Threshold Criteria

**PSYCHOSIS THRESHOLD/ANTI-PSYCHOTIC TREATMENT THRESHOLD**

| | **YES** | **NO** |
|---|---|---|
| • **Severity Scale Score of 6** on *Unusual Thought Content* subscale, **6** on *Non-Bizarre Ideas*, **5 or 6** on *Perceptual Abnormalities* subscale **and/or 6** on *Disorganized Speech* subscales of the CAARMS | ☐ | ☐ |
| **PLUS**<br>• **Frequency Scale Score of greater than or equal to 4** on *Unusual Thought Content*, *Non-Bizarre Ideas*, *Perceptual Abnormalities* **and/or** *Disorganized Speech* subscales | ☐ | ☐ |
| **PLUS**<br>• Symptoms present for **longer than one week** | ☐ | ☐ |
| **PSYCHOSIS THRESHOLD CRITERION MET** | ☐ | ☐ |

From: Yung, L. Phillips, M.B. Simmons, J. Ward, K. Thompson, P. French, P. McGorry (2015). Comprehensive Assessment of At Risk Mental States (CAARMS) – Brief Version. Accessed at: https://www.rcpsych.ac.uk/docs/default-source/improving-care/ccqi/quality-networks/early-intervention-in-psychosis-teams-(eipn)/eipn-brief-caarms-with-sofas-2016.pdf?sfvrsn=49c0749e_2

©2019 Modinos G et al. *JAMA Psychiatry*.

**eTable 5.** Number of Participants by Group in the Different Samples Across Scanners That Had MRI and DFAR Data

| **Baseline** | | | | | | | | | | |
|---|---|---|---|---|---|---|---|---|---|---|
| | **Amsterdam_1 N=28** | **Amsterdam_2 N=42** | **Basel N=20** | **Cologne N=7** | **Copenhagen N=17** | **London N=102** | **Melbourne N=23** | **Paris N=19** | **Vienna N=7** | **Total N=265** |
| HC | 0 | 9 | 0 | 0 | 0 | 36 | 7 | 0 | 0 | 52 |
| CHR | 28 | 33 | 20 | 7 | 17 | 66 | 16 | 19 | 7 | 213 |
| **Within CHR: 12 months overall functioning outcomes** | | | | | | | | | | |
| | **Amsterdam_1 N=21** | **Amsterdam_2 N=26** | **Basel N=14** | **Cologne N=5** | **Copenhagen N=16** | **London N=41** | **Melbourne N=6** | **Paris N=1** | **Vienna -** | **Total N=130** |
| CHR-GO | 6 | 11 | 5 | 1 | 5 | 10 | 1 | 0 | - | 39 |
| CHR-PO | 15 | 15 | 9 | 4 | 11 | 31 | 5 | 1 | - | 91 |
| **Within CHR: 12 months transition / non-transition outcomes** | | | | | | | | | | |
| | **Amsterdam_1 N=28** | **Amsterdam_2 N=33** | **Basel N=20** | **Cologne N=7** | **Copenhagen N=17** | **London N=66** | **Melbourne N=16** | **Paris N=19** | **Vienna N=7** | **Total N=213** |
| CHR-NT | 25 | 32 | 17 | 3 | 13 | 51 | 12 | 12 | 4 | 169 |
| CHR-T | 3 | 1 | 3 | 4 | 4 | 15 | 4 | 7 | 3 | 44 |

Scanner: Amsterdam_1 = 3T Phillips Intera; Amsterdam_2 = 3T Phillips Ingenia; Basel = 3T Siemens Magnetom Verio; Cologne = 3T Siemens Magnetom TrioTim; Copenhagen = 3T Phillips Achieva; London = 3T GE Signa HDx; Melbourne = 3T Siemens Magnetom TrioTim; Paris = 3T Siemens Magnetom TrioTim; Vienna = 3T Siemens Magnetom TrioTim.

Head coil: Amsterdam_1 = Philips 8 Channel SENSE Head Coil; Amsterdam_2 = information not available; Basel = Siemens 12-channel head coil; Cologne = Siemens Head Matrix 12 channel coil; Copenhagen = 8-channel SENSE head coil; London = 8HR BRAIN MRI head coil; Melbourne = Siemens 32-channel head coil; Paris = 12-channel head coil; Vienna = Siemens 12-channel head coil.

Note: Participants recruited in the Amsterdam and The Hague sites were all scanned in the Amsterdam imaging center, which changed scanners halfway through the study; hence the Amsterdam1 and Amsterdam2 nomenclature.

**eTable 6.** DFAR Performance by Site and GMV by Scanner

**DFAR Performance**

| | **Amsterdam_1 N=16** | | **The Hague N=54** | | **Basel N=20** | **Cologne N=7** | **Copenhagen N=17** | **London N=102** | | **Melbourne N=23** | | **Paris N=19** | **Vienna N=7** | **Total N=265** | |
|---|---|---|---|---|---|---|---|---|---|---|---|---|---|---|---|
| | HC (5) | CHR (11) | HC (4) | CHR (50) | CHR | CHR | CHR | HC (36) | CHR (66) | HC (7) | CHR (16) | CHR | CHR | HC (52) | CHR (213) |
| Neutral | 14.0 (2.4) | 13.0 (1.5) | 13.8 (1.7) | 12.6 (2.9) | 12.9 (2.5) | 13.9 (2.0) | 13.6 (2.1) | 13.2 (2.4) | 12.7 (2.4) | 11.1 (1.5) | 12.9 (1.7) | 13.2 (2.0) | 14.0 (2.1) | 13.0 (2.4) | 12.9 (2.4) |
| Happy | 13.6 (1.5) | 15.3 (1.3) | 14.0 (1.4) | 14.3 (2.4) | 14.5 (1.4) | 13.7 (2.4) | 15.5 (0.8) | 14.9 (1.2) | 14.2 (2.4) | 13.4 (2.1) | 14.2 (2.0) | 15.6 (0.8) | 15.3 (0.8) | 14.5 (1.4) | 14.6 (2.1) |
| Fear | 7.8 (1.6) | 11.3 (3.4) | 11.8 (1.0) | 9.9 (2.9) | 9.2 (3.3) | 10.1 (2.9) | 10.9 (2.5) | 9.9 (3.2) | 8.7 (3.0) | 10.4 (2.8) | 10.6 (2.5) | 9.8 (3.0) | 9.1 (2.0) | 9.9 (3.0) | 9.6 (3.0) |
| Angry | 10.6 (1.1) | 11.8 (2.1) | 10.3 (3.6) | 11.8 (3.7) | 12.8 (2.9) | 11.7 (4.2) | 11.7 (2.6) | 11.6 (3.5) | 11.2 (3.8) | 10.1 (2.8) | 11.0 (3.3) | 12.1 (3.0) | 9.6 (4.5) | 11.2 (3.2) | 11.6 (3.5) |

**GMV**

| | **Amsterdam_1 N=28** | **Amsterdam_2 N=42** | **Basel N=20** | **Cologne N=7** | **Copenhagen N=17** | **London N=102** | **Melbourne N=23** | **Paris N=19** | **Vienna N=7** | **Total N=265** |
|---|---|---|---|---|---|---|---|---|---|---|
| GMV | 0.738 (0.090) | 0.750 (0.065) | 0.757 (0.061) | 0.769 (0.142) | 0.745 (0.078) | 0.786 (0.078) | 0.746 (0.067) | 0.744 (0.062) | 0.714 (0.075) | 0.762 (0.078) |

Note: Participants recruited in the Amsterdam and The Hague sites were all scanned in the Amsterdam imaging center, which changed scanners halfway through the study; hence the Amsterdam1 and Amsterdam2 nomenclature for 'scanner'.

©2019 Modinos G et al. *JAMA Psychiatry*.

**eTable 7**. Group Differences in Facial Emotion Recognition (Adjusted for Age, Sex, IQ, Site and General Facial Recognition) Excluding the Sites That Did Not Contribute HC Data

| **DFAR** | **CHR-GO (N=39)** | **CHR-PO (N=90)** | **OR** | **P** | **95% CI** |
|---|---|---|---|---|---|
| Neutral | 13.1 (2.1) | 12.8 (2.3) | 1.03 | 0.77 | 0.85-1.24 |
| Happy | 14.6 (1.5) | 14.6 (2.0) | 0.96 | 0.70 | 0.76-1.21 |
| Fear | 10.4 (3.0) | 9.6 (3.0) | 1.13 | 0.13 | 0.96-1.32 |
| Anger | 11.0 (3.9) | 12.0 (3.4) | 0.88 | **0.03** | 0.78-0.99 |

CHR-GO: clinical high risk good outcome (GAF≥65), CHR-PO: clinical high risk poor outcome (GAF<65), CI, confidence intervals, OR: odds ratio.

©2019 Modinos G et al. *JAMA Psychiatry*.

**eTable 8.** Group Differences in Facial Emotion Recognition (Adjusted for Age, Sex, IQ, Site and General Facial Recognition) Excluding the Site Contributing One Participant to the CHR-GO vs CHR-PO Analysis

| **DFAR** | **HC (N=52)** | **CHR (N=115)** | **OR** | **P** | **95% CI** |
|---|---|---|---|---|---|
| Neutral | 13.0 (2.4) | 12.8 (2.4) | 1.02 | 0.79 | 0.86-1.22 |
| Happy | 14.5 (1.4) | 14.2 (2.4) | 0.99 | 0.91 | 0.81-1.21 |
| Fear | 9.9 (3.0) | 9.5 (3.1) | 0.88 | 0.10 | 0.76-1.02 |
| Anger | 11.2 (3.2) | 11.5 (3.7) | 1.09 | 0.18 | 0.96-1.23 |

CHR: clinical high risk, CI, confidence intervals, HC: healthy controls, OR: odds ratio.

©2019 Modinos G et al. *JAMA Psychiatry*.

**eTable 9.** Group Differences in Facial Emotion Recognition Associated with Longitudinal Outcomes Adjusted for Age, Sex, IQ, Site and General Facial Recognition and Baseline Levels of Prodromal Symptoms (CAARMS Positive) or Global Functioning (GAF)

| **DFAR** | **CHR-GO (N=39) vs CHR-PO (N=91) adjusted by baseline GAF scores** | | | **CHR-GO (N=39) vs CHR-PO (N=91) adjusted by clinical outcomes** | | | **CHR-NT (N=169) vs CHR-T (N=44) adjusted by baseline CAARMS Positive scores** | | |
|---|---|---|---|---|---|---|---|---|---|
| | OR | P | 95% CI | OR | P | 95% CI | OR | P | 95% CI |
| Neutral | 1.04 | 0.68 | 0.86-1.26 | 1.03 | 0.80 | 0.84-1.25 | 0.91 | 0.29 | 0.77-1.08 |
| Happy | 0.97 | 0.80 | 0.77-1.23 | 0.92 | 0.51 | 0.71-1.18 | 1.02 | 0.83 | 0.84-1.25 |
| Fear | 1.11 | 0.21 | 0.94-1.31 | 1.13 | 0.14 | 0.93-1.34 | 0.99 | 0.84 | 0.85-1.14 |
| Anger | 0.88 | **0.03** | **0.78-0.99** | 0.87 | **0.02** | **0.77-0.98** | 0.99 | 0.92 | 0.89-1.11 |

CHR-GO: clinical high risk good outcome (GAF≥65), CHR-NT: clinical high risk nontransition, CHR-PO: clinical high risk poor outcome (GAF<65), CHR-T: clinical high risk transition, CI, confidence intervals, OR: odds ratio.

©2019 Modinos G et al. *JAMA Psychiatry*.

**eTable 10.** Analysis of Potential Confounders on GMV-DFAR Interactions Observed With Baseline Status (HC, CHR) and Functional Outcomes (CHR-GO, CHR-PO)

| | **Antidepressants (yes/no)** | | **Cigarettes/ day** | | **Alcohol/ day** | | **Cannabis (yes/no)** | | **CAARMS Anxiety** | | **CAARMS Depression** | |
|---|---|---|---|---|---|---|---|---|---|---|---|---|
| | **F** | **P** | **r** | **P** | **r** | **P** | **F** | **P** | **r** | **P** | **r** | **P** |
| **From HC vs CHR DFAR-GMV interaction** | | | | | | | | | | | | |
| **HC** | | | | | | | | | | | | |
| DFAR Happy: left MPFC | N/A | N/A | -.251 | .21 | -.005 | .97 | .509 | .48 | .013 | .93 | -.124 | .39 |
| DFAR Anger: MPFC | N/A | N/A | -.098 | .63 | .152 | .33 | .045 | .83 | -.104 | .47 | .183 | .20 |
| **CHR** | | | | | | | | | | | | |
| DFAR Happy: left MPFC | 3.492 | .06 | -.060 | .48 | .009 | .91 | 1.357 | .25 | -.054 | .44 | .001 | .99 |
| DFAR Anger: MPFC | **8.225** | **.005** | .164 | .06 | .048 | .55 | .001 | .97 | -.015 | .83 | -.051 | .46 |
| **From CHR-GO vs CHR-PO DFAR-GMV interaction** | | | | | | | | | | | | |
| **CHR-GO** | | | | | | | | | | | | |
| DFAR Anger: left hippocampus | .488 | .49 | -.038 | .85 | -.109 | .55 | .078 | .78 | .128 | .44 | -.025 | .88 |
| DFAR Fear: left MPFC | .277 | .60 | .065 | .75 | .085 | .64 | 1.414 | .24 | .051 | .76 | .155 | .35 |
| **CHR-PO** | | | | | | | | | | | | |
| DFAR Anger: left hippocampus | 1.615 | .21 | -.034 | .81 | .135 | .27 | .559 | .46 | .219 | .04 | .031 | .77 |
| DFAR Fear: left MPFC | .039 | .84 | .012 | .93 | .004 | .97 | 3.292 | .07 | .074 | .50 | .000 | .99 |

Cigarettes, alcohol, and CAARMS variables: Pearson's product-moment correlation. Cannabis: one-way ANOVA between participants who endorsed smoking cannabis (HC: 15, CHR: 62, CHR-GO: 11, CHR-PO: 31) compared to those who did not (HC: 21, CHR: 110, CHR-GO: 23, CHR-PO: 45). Antidepressants: one-way ANOVA between participants who were on antidepressants (CHR: 65, CHR-GO: 9, CHR-PO: 30) compared to those who were not (CHR: 102, CHR-GO: 22, CHR-PO: 42).

©2019 Modinos G et al. *JAMA Psychiatry*.

**eFigure 1.** Group x GMV x DFAR Interactions After Removing One Participant With a Low BFRT Score (Covarying for Age, Sex, Scanner, IQ and BRFT) ($p_{FWE}$<0.05)

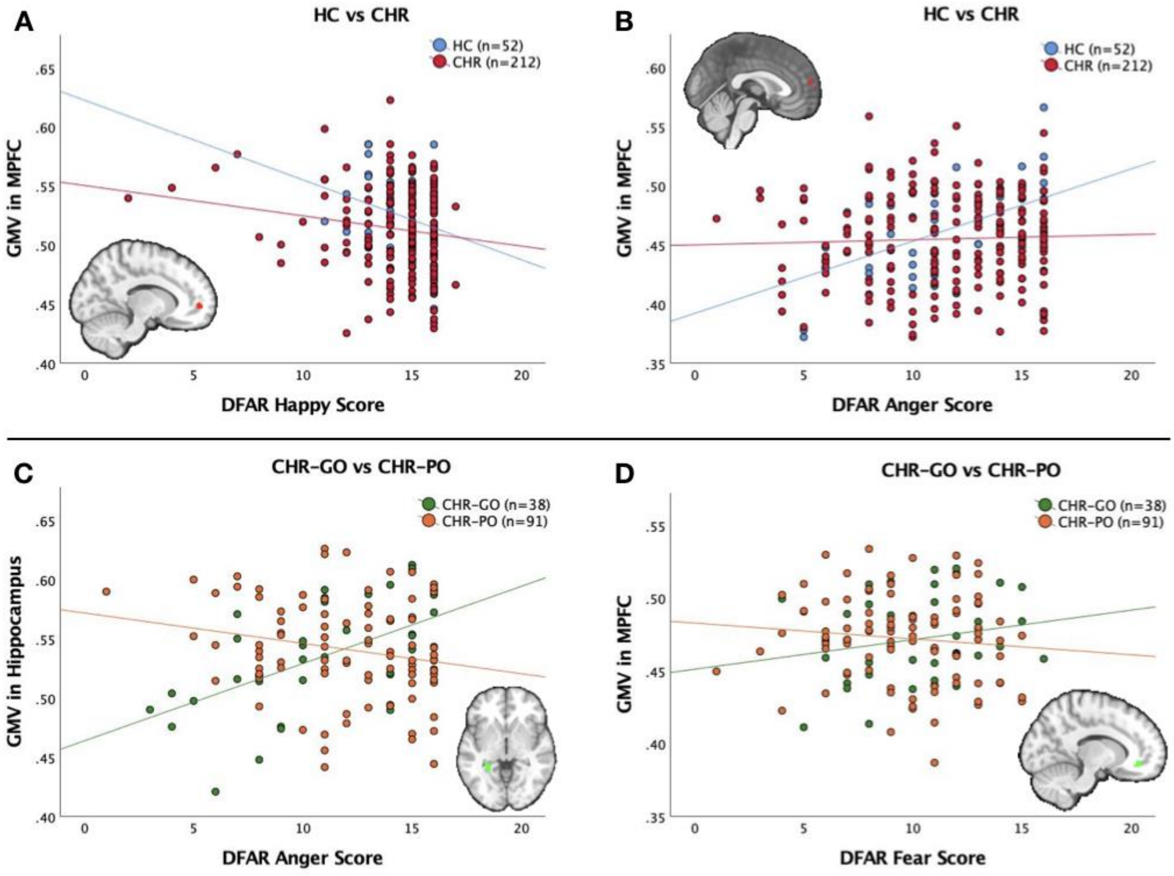


©2019 Modinos G et al. *JAMA Psychiatry*.

**eFigure 2.** QA Results from the Application of CAT12 to the Structural MRI Images of the 265 Study Participants

The violin plot displays the homogeneity of our sample (correlation values for each subject) as calculated by CAT12 applied to the modulated normalized grey matter images. The two volumes with a relatively low rating where checked again carefully; no artefacts were identified.

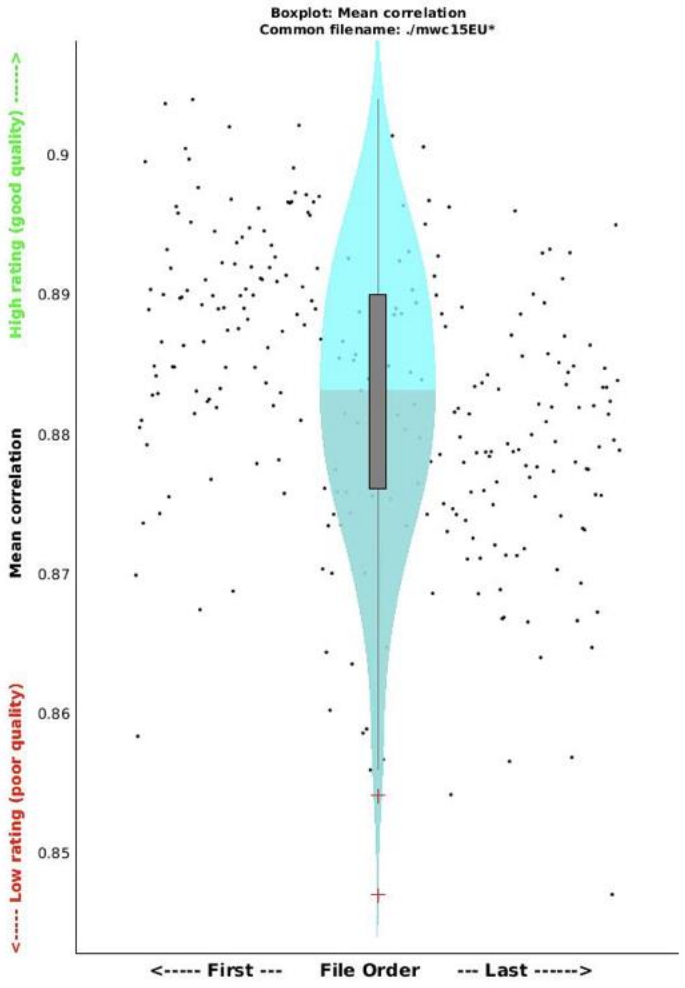


©2019 Modinos G et al. *JAMA Psychiatry*.

**eFigure 3.** Partial Correlation Plots of the Associations Between Baseline DFAR Performance and GAF Change Scores (Adjusted for Age, Sex, IQ, Site and BFRT Score)

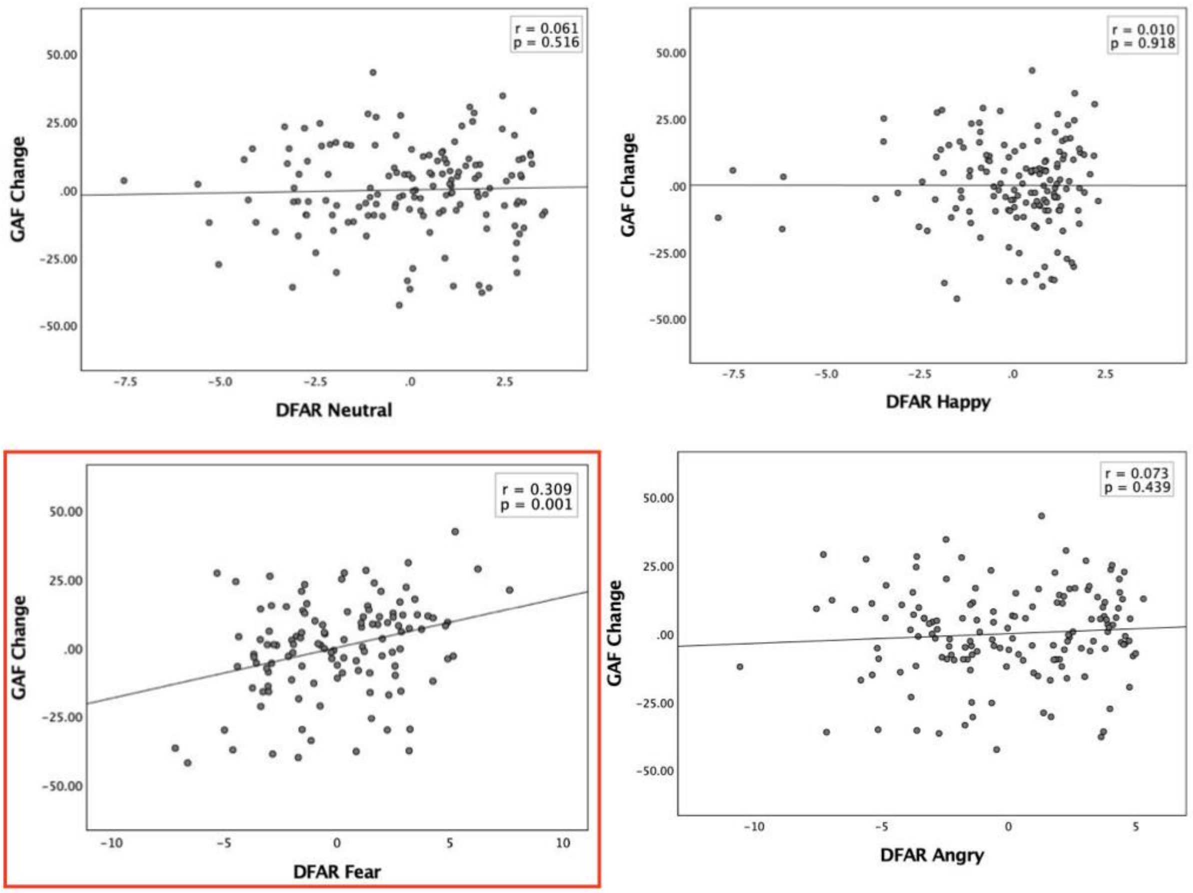


©2019 Modinos G et al. *JAMA Psychiatry*.

**eFigure 4**. Group x GMV x DFAR Interactions After Excluding the Sites That Did Not Contribute HC Data (Covarying for Age, Sex, Scanner, IQ and BRFT) ($p_{FWE}$<0.05)

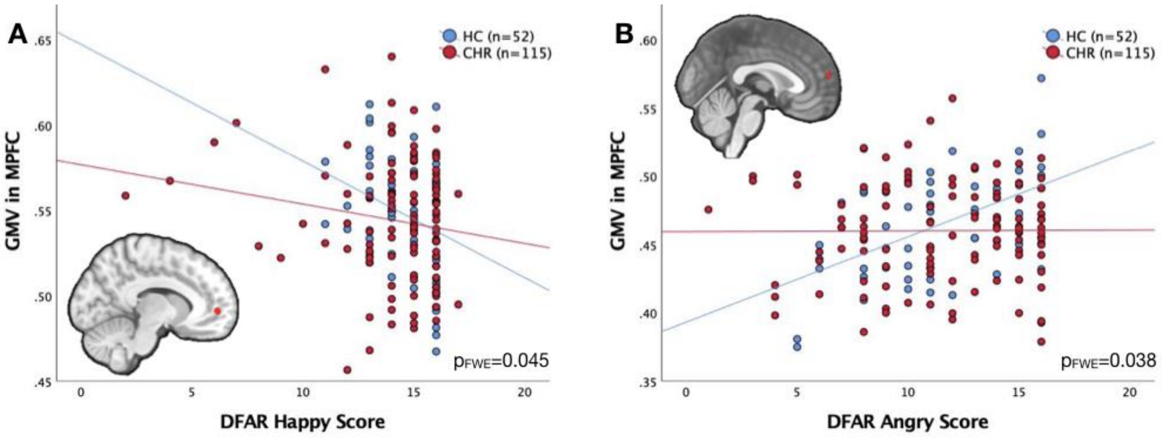


©2019 Modinos G et al. *JAMA Psychiatry*.

**eFigure 5**. Group x GMV x DFAR Interactions After Removing the Site Contributing One Participant to the CHR-GO vs CHR-PO Analysis (Covarying for Age, Sex, Scanner, IQ and BRFT) ($p_{FWE}$<0.05)

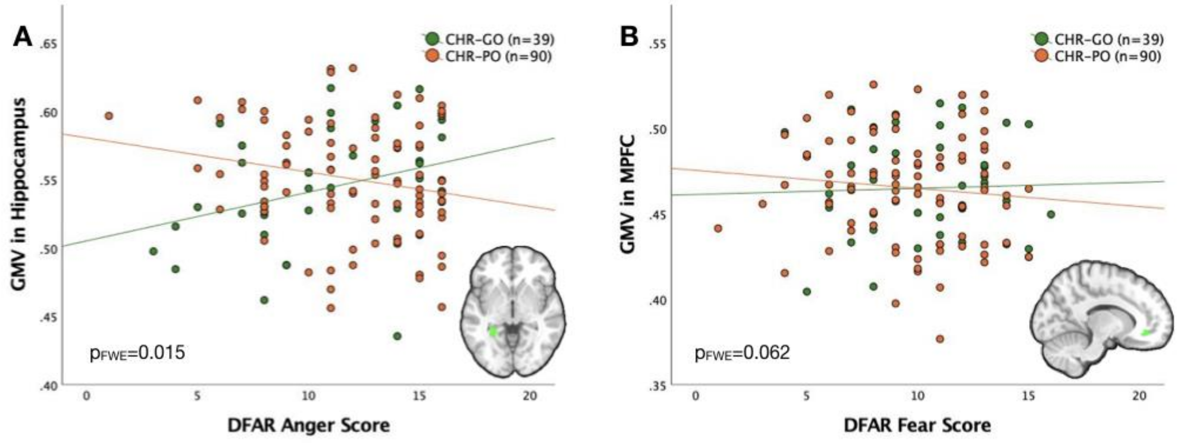


©2019 Modinos G et al. *JAMA Psychiatry*.

**eFigure 6.** Analysis of the Distribution of CHR-NT/CHR-T Individuals in the CHR-GO/CHR-PO Follow-up Groups

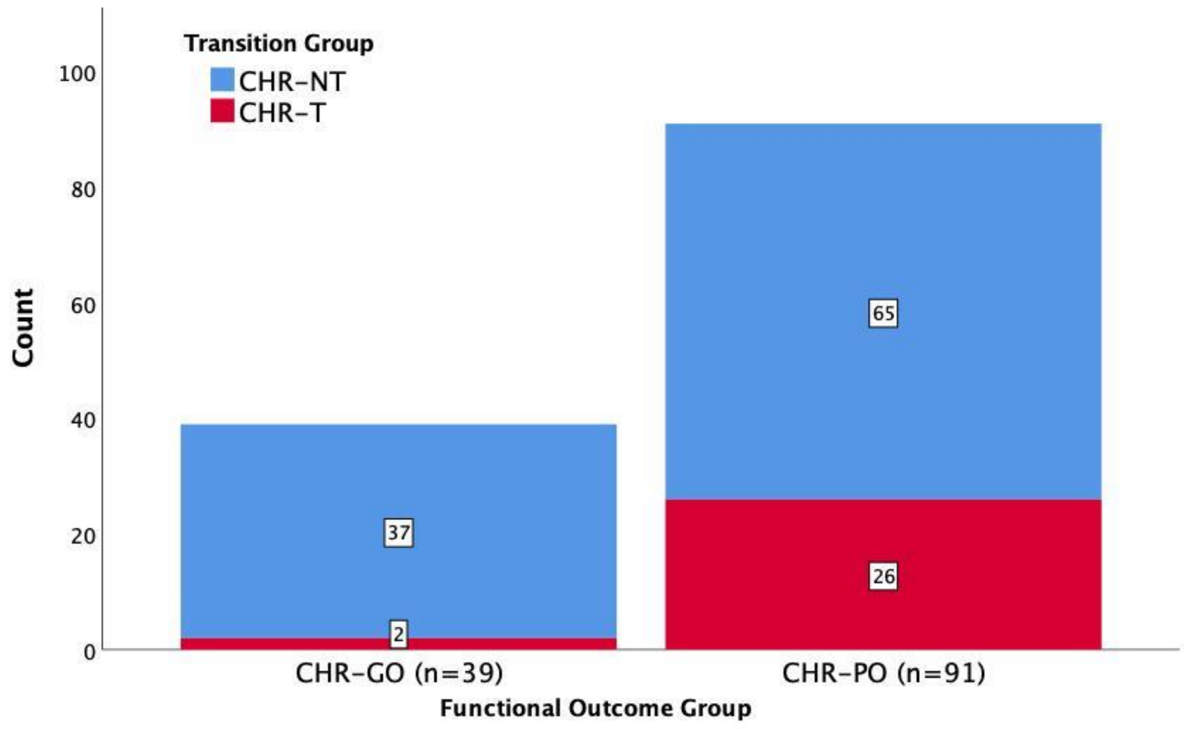


©2019 Modinos G et al. *JAMA Psychiatry*.

**eFigure 7.** Baseline Status x GMV x DFAR Interactions After Removing CHR Participants (n=20) Treated With Antipsychotics (Covarying for Age, Sex, Scanner, IQ and BRFT) ($p_{FWE}$<0.05)

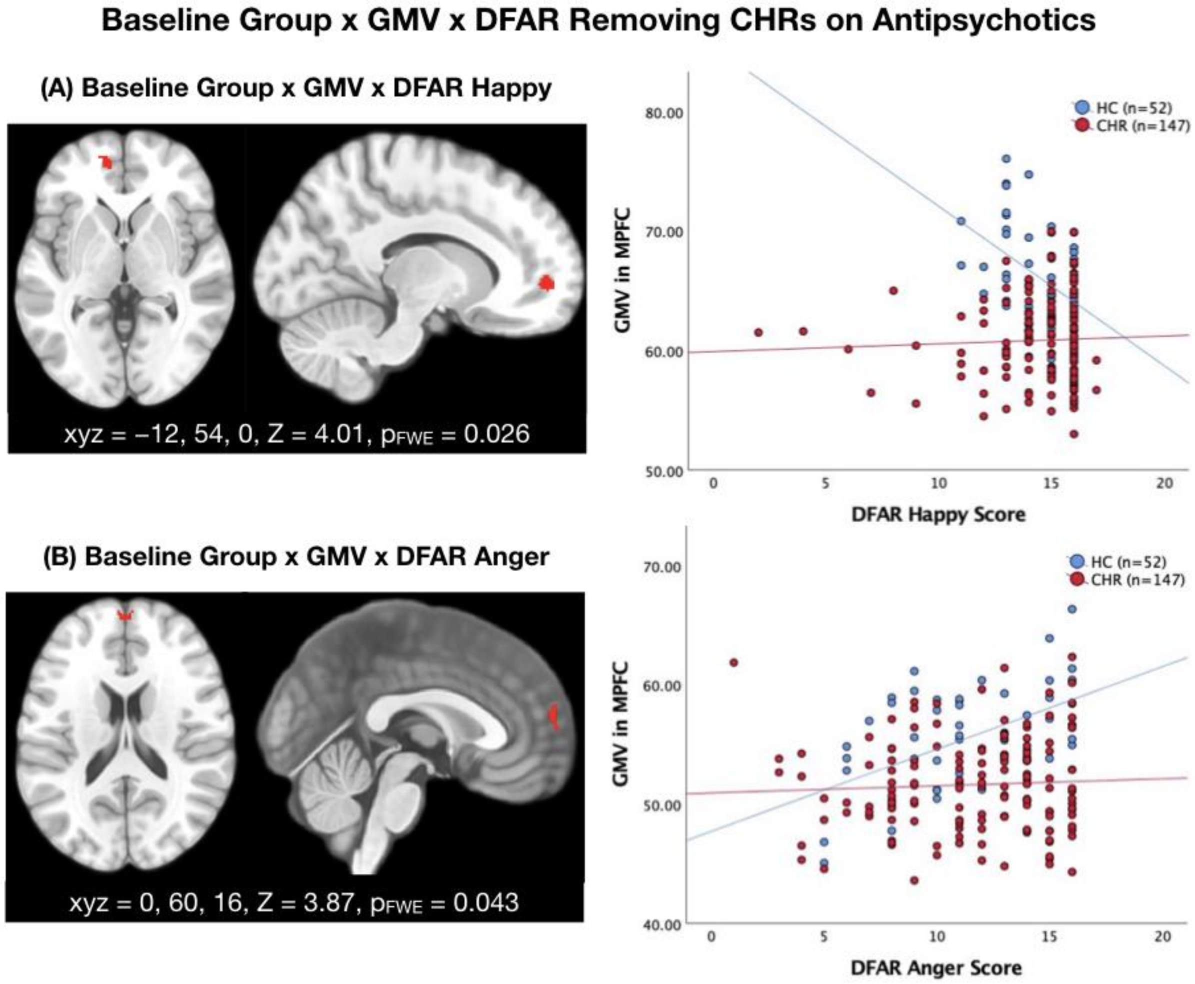


©2019 Modinos G et al. *JAMA Psychiatry*.

**eFigure 8.** Functional Outcome x GMV x DFAR Interactions After Removing CHR Participants (n=11) Treated With Antipsychotics (Covarying for Age, Sex, Scanner, IQ and BRFT) ($p_{FWE}$<0.05)

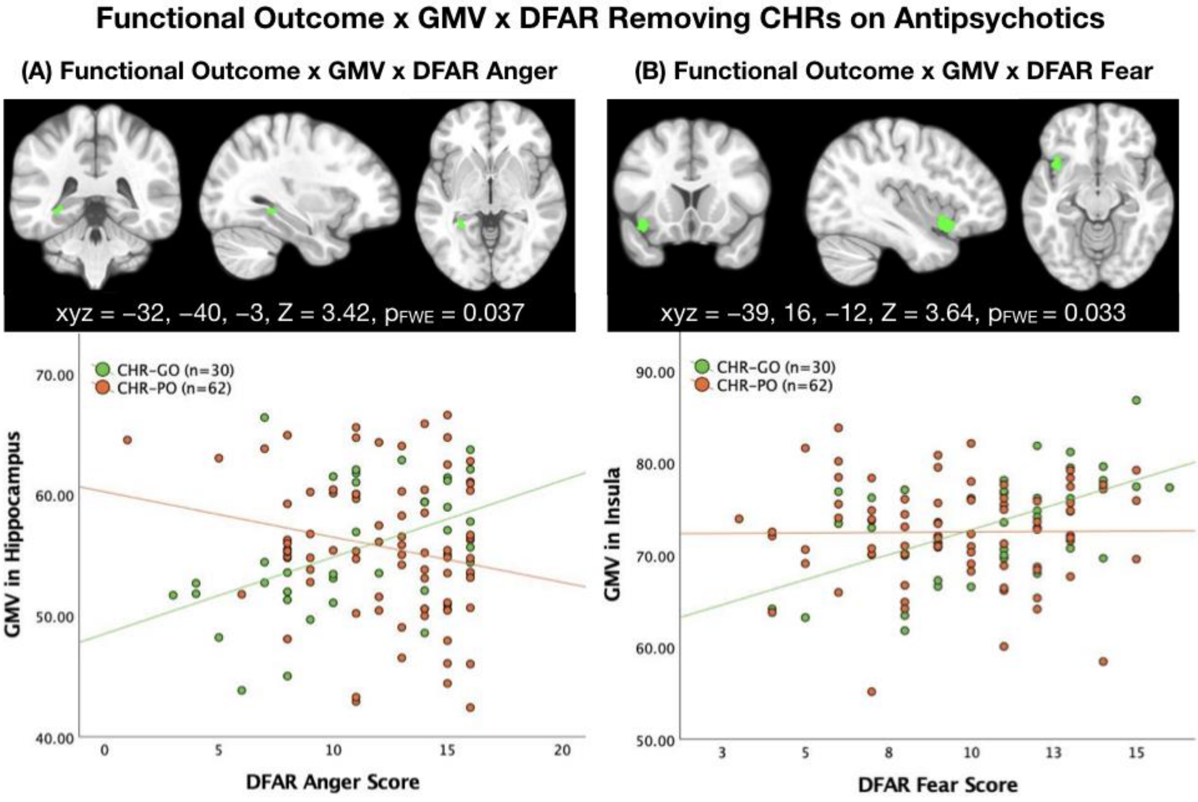


©2019 Modinos G et al. *JAMA Psychiatry*.

**eFigure 9.** Functional Outcome x GMV x DFAR Interactions Covarying for Age, Sex, Scanner, IQ, BRFT and Baseline GAF Scores ($p_{FWE}<0.05$)

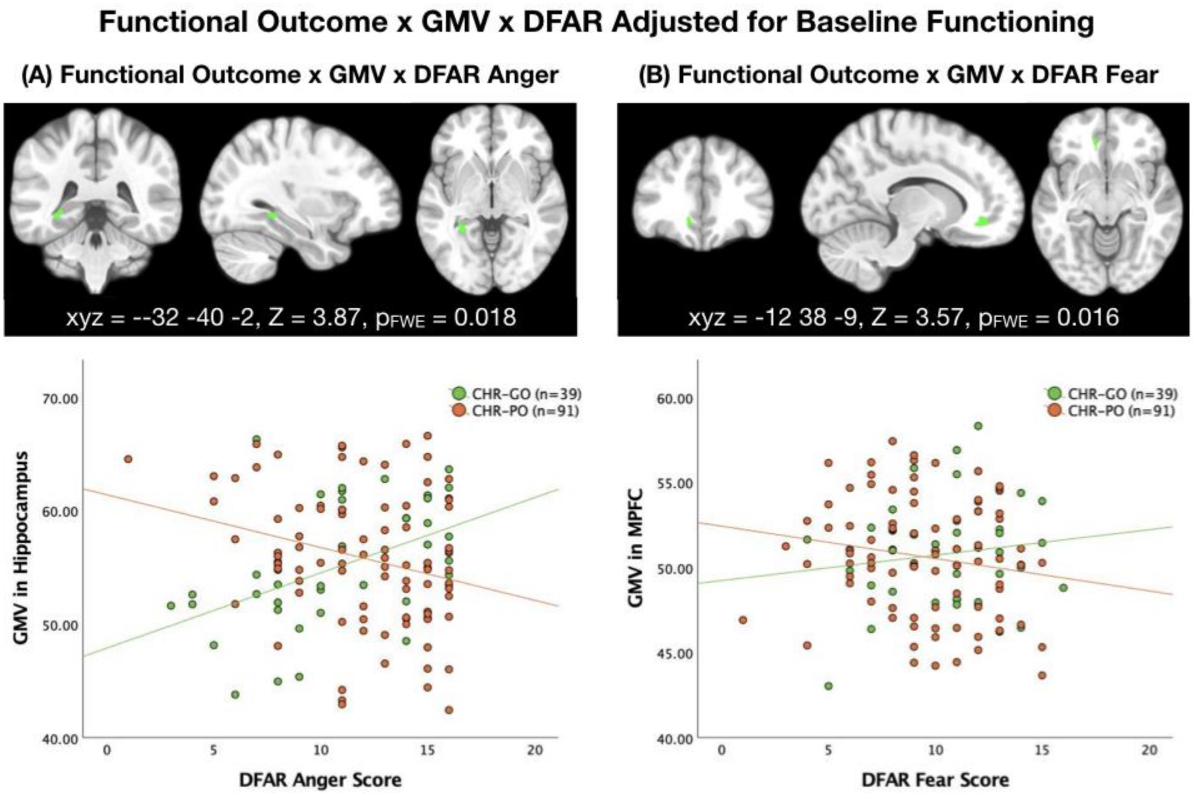


©2019 Modinos G et al. *JAMA Psychiatry*.

**eReferences**


1. Ashburner J. VBM Tutorial. *Available at: https://wwwfilionuclacuk/~john/misc/VBMclass15pdf.* 2015.
2. Levin HS, Hamsher KdS, Benton AL. A Short Form of the Test of Facial Recognition for Clinical Use. *The Journal of Psychology.* 1975;91(2):223-228.
3. Brett M, Anton JL, Valabregue R, Poline JB. Region of interest analysis using an SPM toolbox [abstract]. Paper presented at: 8th International Conference on Functional Mapping of the Human Brain2002; Sendai, Japan.


©2019 Modinos G et al. *JAMA Psychiatry*.
